# Identification of Novel Chemical Scaffolds Inhibiting Trypanothione Synthetase from Pathogenic Trypanosomatids

**DOI:** 10.1371/journal.pntd.0004617

**Published:** 2016-04-12

**Authors:** Diego Benítez, Andrea Medeiros, Lucía Fiestas, Esteban A. Panozzo-Zenere, Franziska Maiwald, Kyriakos C. Prousis, Marina Roussaki, Theodora Calogeropoulou, Anastasia Detsi, Timo Jaeger, Jonas Šarlauskas, Lucíja Peterlin Mašič, Conrad Kunick, Guillermo R. Labadie, Leopold Flohé, Marcelo A. Comini

**Affiliations:** 1 Laboratory Redox Biology of Trypanosomes, Institut Pasteur de Montevideo, Montevideo, Uruguay; 2 Departamento de Bioquímica, Universidad de la República, Montevideo, Uruguay; 3 Instituto de Química Rosario-CONICET, Facultad de Ciencias Bioquímicas y Farmacéuticas, Universidad Nacional de Rosario, Rosario, Argentina; 4 Institut für Medizinische und Pharmazeutische Chemie, Technische Universität Braunschweig, Braunschweig, Germany; 5 Institute of Biology, Medicinal Chemistry and Biotechnology, National Hellenic Research Foundation, Athens, Greece; 6 Laboratory of Organic Chemistry, School of Chemical Engineering, National Technical University of Athens, Athens, Greece; 7 German Centre for Infection Research, Braunschweig, Germany; 8 Department of the Biochemistry of Xenobiotics Institute of Biochemistry, Vilnius University, Vilnius, Lithuania; 9 Department for Medicinal Chemistry, Faculty of Pharmacy, University of Ljubljana, Ljubljana, Slovenia; 10 Department of Molecular Medicine, Università degli Studi di Padova, Padova, Italy; Northeastern University, UNITED STATES

## Abstract

**Background:**

The search for novel chemical entities targeting essential and parasite-specific pathways is considered a priority for neglected diseases such as trypanosomiasis and leishmaniasis. The thiol-dependent redox metabolism of trypanosomatids relies on bis-glutathionylspermidine [trypanothione, T(SH)_2_], a low molecular mass cosubstrate absent in the host. In pathogenic trypanosomatids, a single enzyme, trypanothione synthetase (TryS), catalyzes trypanothione biosynthesis, which is indispensable for parasite survival. Thus, TryS qualifies as an attractive drug target candidate.

**Methodology/Principal Finding:**

A library composed of 144 compounds from 7 different families and several singletons was screened against TryS from three major pathogen species (*Trypanosoma brucei*, *Trypanosoma cruzi* and *Leishmania infantum*). The screening conditions were adjusted to the TryS´ kinetic parameters and intracellular concentration of substrates corresponding to each trypanosomatid species, and/or to avoid assay interference. The screening assay yielded suitable Z’ and signal to noise values (≥0.85 and ~3.5, respectively), and high intra-assay reproducibility. Several novel chemical scaffolds were identified as low μM and selective tri-tryp TryS inhibitors. Compounds displaying multi-TryS inhibition (*N*,*N'*-bis(3,4-substituted-benzyl) diamine derivatives) and an *N*^*5*^-substituted paullone (MOL2008) halted the proliferation of infective *Trypanosoma brucei* (EC_50_ in the nM range) and *Leishmania infantum* promastigotes (EC_50_ = 12 μM), respectively. A bis-benzyl diamine derivative and MOL2008 depleted intracellular trypanothione in treated parasites, which confirmed the on-target activity of these compounds.

**Conclusions/Significance:**

Novel molecular scaffolds with on-target mode of action were identified as hit candidates for TryS inhibition. Due to the remarkable species-specificity exhibited by tri-tryp TryS towards the compounds, future optimization and screening campaigns should aim at designing and detecting, respectively, more potent and broad-range TryS inhibitors.

## Author Summary

Parasites from the genus *Trypanosoma* and *Leishmania* are etiologic agents for a group of neglected diseases with high morbidity and mortality rates in the developing world. Inasmuch as vaccine development is hampered by the successful mechanisms employed by the pathogens to evade the host immune response, chemotherapy remains as a safe option to fight these diseases. However, new drugs with better pharmacological performance (i.e. safety, efficacy and ease of administration) than those in current use are urgently needed. The thiol-redox metabolism of trypanosomatids offers an excellent opportunity for the development of more selective and efficacious medicines because it depends on a molecule, trypanothione (a bis-glutathionyl derivative of spermidine), unique and indispensable to the pathogens. Here we report the identification of novel inhibitors of trypanothione synthetase from three major trypanosomatid species of medical and veterinary relevance. Although highly conserved in sequence, trypanothione synthetases display significant species-specifity towards compounds, pointing to structural differences as determinants of ligand selectivity. Most of the active compounds presented two-digit μM inhibitory activity and serve as primary scaffolds to develop more potent inhibitors. Among them, *N*,*N'*-bis(benzyl)-substituted diamine and paullone derivatives are interesting candidates because of their potent and/or selective anti-trypanosomal and anti-trypanothione synthetase activity.

## Introduction

Protozoan parasites from the genus *Trypanosoma* and *Leishmania* are responsible for diseases affecting humans and their livestock. The zoonotic character of these diseases, which involve different insect species as vectors and wild animals as reservoirs, hamper the implementation of successful control strategies [1]. Immuno-prophylaxis is not yet available and for some species, such as *T*. *brucei* spp. and *T*. *cruzi*, appears unfeasible due to complex immune-evasion mechanisms [2, 3]. So far, and probably for several decades ahead, chemotherapy remains as the sole choice of treatment. Only a handful of drugs are available to fight Chagas’s disease (*T*. *cruzi*), sleeping sickness (*T*. *brucei gambiense* and *T*. *b*. *rhodesiense*) and the different forms of leishmaniasis (*Leishmania spp*.). Unfortunately, they suffer from several drawbacks encompassing low efficacy, resistance and route of administration [4–7]. Moreover, several of these drugs (e.g. nifurtimox, benznidazole and melarsoprol) present a non-specific mode of action that accounts for their high toxicity [8, 9]. Thus, the discovery of new chemical entities targeting specific and indispensable components of parasite metabolism is a priority for trypanosomiasis and leishmaniasis.

**Fig 1:**
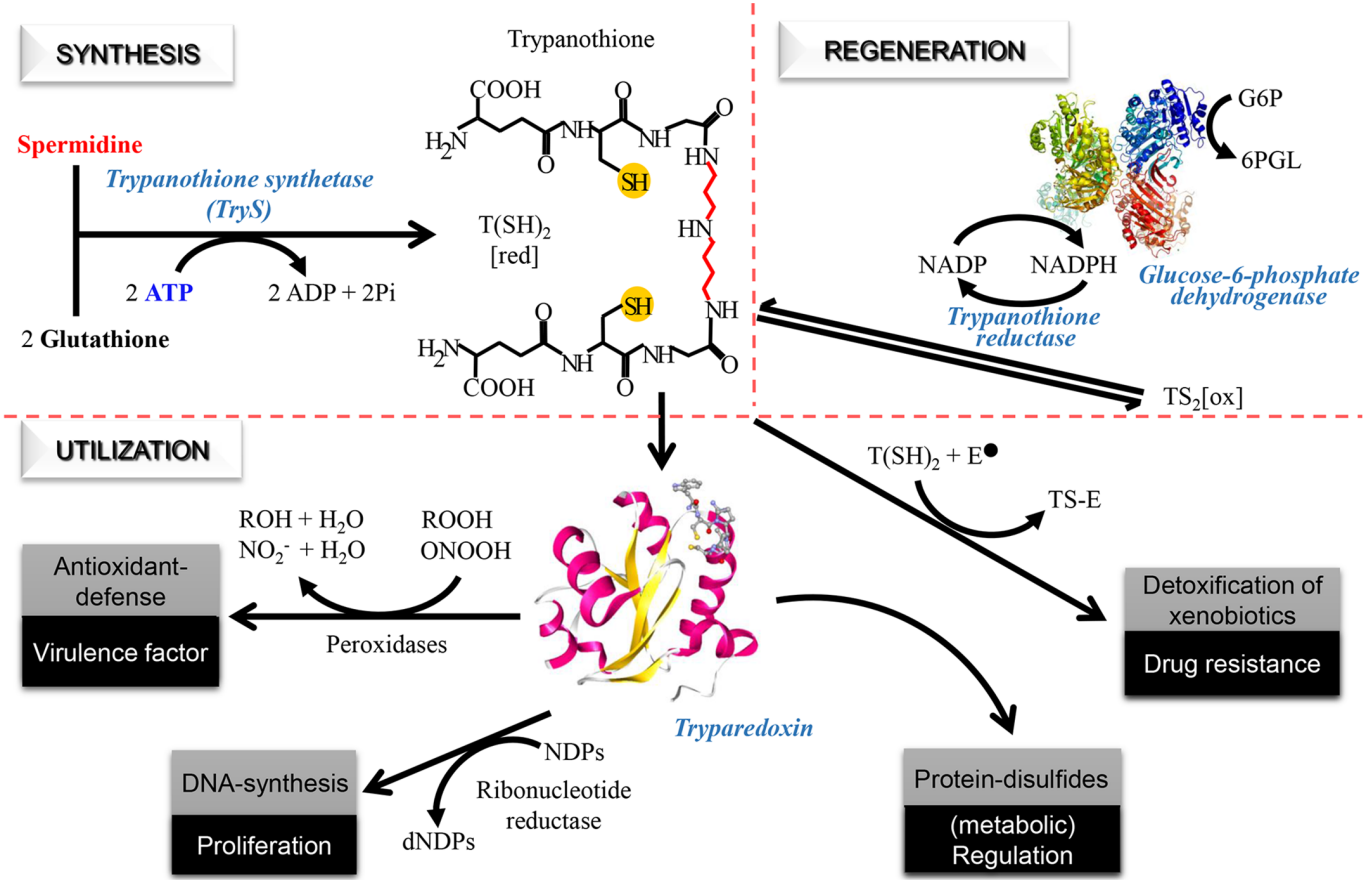
Trypanothione dependent redox metabolism. The chemical structure of trypanothione (*N*^1^,*N*^8^-bis(glutathionyl)spermidine; T(SH)_2_) is depicted at the center. Synthesis: trypanothione synthetase catalyzes the ligation of two molecules of gluthatione to one of spermidine using the energy provided by two ATP molecules. Regeneration: trypanothione reductase maintains trypanothione in the reduced state at expenses of NADPH, which can be supplied by the oxidative phase of the pentose phosphate pathway *via* glucose 6-phosphate dehydrogenase. Utilization: reduced trypanothione is involved in multiple functions such as the detoxification of xenobiotics, cell proliferation, defense against oxidants and protein thiol-redox homeostasis. The multipurpose oxidoreductase tryparedoxin plays an important role catalyzing electron transfer from T(SH)_2_ to different molecular targets (e.g. peroxidases, ribonucleotide reductase and protein disulfides). G6P: glucose-6-phosphate, 6PGL: 6-phosphogluconolactone, T(SH)_2_: reduced trypanothione, TS_2[ox]_: oxidized trypanothione, NDPs: nucleosides diphosphate, dNDP: deoxinucleosides diphosphate, E^-^: electrophilic species, TS-E: trypanothione-electrophile adduct, ROOH: hydroperoxide, ONOOH: peroxynitrite, NO_2_^-^: nitrite.

The thiol-dependent redox metabolism is one of the unique metabolic features that distinguish trypanosomatids from humans and offer reliable molecular targets for selective drug development [10]. An example of genetic hallmarks of trypanosomatids is the lack of genes coding for glutathione reductase and thioredoxin reductase [11–13], which fuel the major redox systems of most living organisms (i.e. the glutathione/glutaredoxin system and the thioredoxin system) with reducing equivalents. Instead, trypanosomatids rely on the low molecular mass thiol *N*^1^, *N*^8^-bis(glutathionyl)spermidine [trypanothione, T(SH)_2_] and the flavoenzyme trypanothione reductase for sustaining the intracellular redox homeostasis (Fig 1). T(SH)_2_ delivers reducing potential to different redoxin proteins, which, by acting on different targets, control vital functions (for a review see [10]; Fig 1). Proposed functions of T(SH)_2_ further comprise neutralization of xeno- and endobiotics (e.g. methylglyoxal, iron and nitric oxide), coordination of iron-sulfur complexes and reduction of ascorbate [14, 15].

The biosynthesis of T(SH)_2_ is achieved in two consecutive steps each involving the ligation of a glutathione (GSH) molecule by its glycine carboxyl group to the free *N*^1^ and *N*^8^ amine groups of spermidine (SP). Both reactions are catalysed by the C-terminal ligase domain of trypanothione synthetase (TryS; EC 6.3.1.9) at the expense of ATP (Fig 1). Some trypanosomatids species harbour (*L*. *infantum*, *L*. *donovani*, *L*. *mexicana* and *T*. *cruzi*) or express (*Crithidia fasciculata*) a gene coding for glutathionylspermidine synthetase (GspS; EC 6.3.1.8), which synthesizes the reaction intermediate *N*^8^ mono-glutathionylspermidine.

The importance of TryS activity for parasite viability has been demonstrated *in vitro* and *in vivo* for *T*. *b*. *brucei* [16–19] and *L*. *infantum* [20] by means of genetic and pharmacological approaches. In addition, TryS presents several advantages as a drug target candidate: (i) it is encoded by a single copy gene [11–13], (ii) the structure of TryS from *L*. *major* has been elucidated [21], (iii) TryS has been shown to provide metabolic control to the trypanothione pathway in *T*. *cruzi* [22], and (iv) kinetic information is available for several TryS [18, 22–27].

At an early state of knowledge, the rational inhibitor design was undertaken using GspS of *C*. *fasciculata* (*Cf*GspS) and *Escherichia coli* (*Ec*GspS) as test enzymes and compounds isosteric with GSH or related transition state analogues as chemical scaffolds [28–34]. Preliminary studies with GSH analogues identified the γ-glutamyl moiety as critical for molecular recognition [28]. Further work revealed that addition of acidic groups to the *L*-γ-Glu-*L*-Leu dipeptide resulted in *Cf*GspS inhibitors of reasonable potency such as the phosphonic (*K*_*i*_ ~ 60 μM) [29], the boronic (*K*i ~ 81 μM and *K*_i_* ~ 18 μM) [34] or the diaminopropionic acid derivative (*K*i ~ 7.2 μM and *K*_i_* ~ 21 μM) [35].

Transition state mimics, previously identified as potent inhibitors of *Ec*GspS [30–32], proved to be equally active against recombinant *Cf*GspS. Notably, a Gsp-phosphinate derivative was capable to inhibit recombinant TryS from *L*. *major*, *T*. *cruzi* and *T*. *b*. *brucei* albeit with apparent *K*_*i*_ values 16–40-fold higher than that obtained for *Cf*GspS (*K*_*i*_ of 18.6 nM) [36]. Unfortunately, these compounds displayed null biological activity against pathogenic trypanosomatids at 100 μM. Nevertheless, this phosphinate remained the only compound able to target TryS from three different pathogenic trypanosomatids.

The anti-proliferative activity of GSH derivates (*N*,*S*-blocked GSH diesters, *S*-2,4-dinitrophenyl-GSH) [37–39] and GSH-related phosphinopeptides [40] ranked from 35 to 0.2 μM in different trypanomatids. The poor biological activity exhibited by these inhibitors has been ascribed to their peptidic nature susceptible to hydrolysis by esterases and amidases [37].

Based on the ATP-dependency of TryS, a compound library of protein kinase inhibitors was screened against recombinant *Cf*TryS. This led to the identification of *N*^5^-acetamide paullones (benzo[2,3]azepino[4,5-*b*]indol-6-ones) as potent inhibitors of *Cf*TryS [10, 41]. Recently, we reported the finding of a related 10-trifluormethylated acetamide derivative of this paullone as nM inhibitor of *Li*TryS (IC_50_ 350 nM) [20]. Information on the activity of these paullones against TryS and parasites from other pathogenic species is lacking.

More recently, a HTS campaign against *Tb*TryS with a library of ~ 62000 compounds identified several hits that upon optimization yielded leaders with inhibitory activity against *Tb*TryS in the nM range (IC_50_ values of 45, 95 and 140 nM for DDU86439, DDD85811 and DDD86243, respectively) and EC_50_ values towards infective *T*. *b*. *brucei* that ranked from 5 to 10 μM. The compounds allowed the chemical validation of *Tb*TryS as drug target [17, 18].

We here describe the setting-up of a screening technique to detect TryS inhibitors and report the identification and chemical validation of inhibitors from TryS from three major trypanosomatid species (*Trypanosoma brucei brucei*: *T*. *b*. *brucei*, *Trypanosoma cruzi*: *T*. *cruzi* and *Leishmania infantum*: *L*. *infantum*).

## Materials and Methods

### Organisms and Reagents

Unless otherwise stated all reagents were of analytical grade and purchased from Sigma-Aldrich, J.T. Baker, Carlo Erba Reagents SA, Gibco, Invitrogen, Life Technologies, Enzo Life Sciencies, Roche.

### Heterologous Expression and Purification of Recombinant TryS

TryS from different trypanosomatids was produced in recombinant form with an N-terminal His-tag. The constructs pET-15b *Tb*TryS [27], pRSET-B *Tc*TryS and pET-28c(+)*Li*TryS [20] were kind gifts of Alan Fairlamb (Dundee University, Dundee, Scotland), Sergio Guerrero (Universidad Nacional del Litoral, Santa Fe, Argentina) and Helena Castro (Institute for Molecular and Cell Biology, Porto, Portugal), respectively. They were used to express TryS of *T*. *b*. *brucei* 427 (MITat1.4, GenBank accession protein id CAC87573.1), *T*. *cruzi* strain Tulahuen 0 (GenBank accession protein id AAO00722.1) and *L*. *infantum* JPCM5 (GenBank accession protein id CAM69145.1). *E*. *coli* strain BL21 (DE3) or Tuner (DE3) (Novagen) served as expression host. For a detailed description of the expression and purification protocols see S1 Text.

Protein concentration was determined using the Bicinconinic Acid assay with bovine serum albumin as standard. The protocols described above yielded 4–8 mg of recombinant TryS per liter of culture medium with ≥ 95% purity and homogeneous specific activity.

### Kinetic Characterization

The kinetic characterization of His-tagged TryS was performed using the LDH/PK assay which couples ATP regeneration to NADH oxidation. The end-point assay based on detection of inorganic phosphate (Pi) by the BIOMOL GREEN reagent was used to estimate the apparent *K*_*i*_ for ADP. All reactions were performed at room temperature (RT, 20–25°C) and a detailed description of both assays is provided in S1 Text. The apparent kinetic parameters (*K*_*M*_ and V_max_) were calculated by fitting plots of initial velocity (*v*) *vs*. substrate concentracion ([S]), determined at saturating concentration of co-substrates, to the Michaelis Menten equation assisted by the software OriginPro 8. For GSH, the *K*_*M*_ and *K*_*i*_ values were determined using the following equation *v* = *V*_*max*_ / (1 + *K*_*M*_ / [GSH] + [GSH] / *K*_*i*_), which considers the nonproductive binding of GSH to the substituted enzyme [42]. The apparent *K*_*i*_ for ADP was estimated from linear fitting of the plots [E]/*v vs*. [ADP] at different concentrations of ATP, where [E] is enzyme concentration.

### Compound Library

The compound library involves 144 chemical entities that are clustered by chemical scaffold as follow: **(A)** 6-arylpyrido[2,3-*d*]pyrimidine-2,7-diamine derivatives (**APPDA**; S1 Table) developed as ATP-competitive inhibitors of bacterial D-Alanine:D-Alanine ligase [43] and biotin carboxylase [44]; **(B)** 1-(benzo[*d*]thiazol-2-yl)-4-benzoyl-3-hydroxy-5-phenyl-1*H*-pyrrol-2(5*H*)-one derivatives (**BBHPP**; S2 Table); **(C)** *N*,*N'*-bis(3,4-substituted-benzyl) diamine derivatives (**BDA**; S3 Table) that display potent anti-malarial or -trypanosomal/leishmanial activity [45, 46] and are simplified derivatives of compounds interfering with the parasite’s polyamine metabolism [45, 47–50]; **(D)** benzofuroxan (**BZ**; S4 Table) [51–55]; **(E)** 4,5-dihydroazepino[4,5-*b*]indol-2(1*H*,3*H*,6*H*)-one derivatives (**AI**; S5 Table), some of which with reported anti-TryS or -*T*. *b*. *brucei* activity [56]; **(F)** 1*H*-purine-2,6(3*H*,7*H*)-dione (**PD**), including the 3-butyl-7-methyl-8-((3-(trifluoromethyl) phenylthio)methyl)3,4,5,7-tetrahydro-1*H*-2,6-dione) (kindly provided by Dr. Luise Krauth-Siegel, Heidelberg University, Germany; S6 Table) that was reported as inhibitor of the T(SH)_2_-dependent oxidoreductase tryparedoxin [57]; **(G)** 2-aminooxazole-5-carboxamide derivatives (**AOCA**; S7 Table); **(H)** and several singletons: *N*,*N*-dibenzyl-1-ethyl-3-methyl-1*H*-pyrazole-5-carboxamide; 2-amino-*N*,*N*-dibenzyl-4-methyl thiazole-5-carboxamide; prochlorperazine; *tert*-butyl 8-aminooctylcarbamate; *tert*-butyl 12-aminododecylcarbamate; *N*-(8-aminooctyl)acetamide.

### TryS Screening Assay Development

#### General considerations

The BIOMOL GREEN assay was adapted for the screening of potential TryS inhibitors. All assays (screening and interference test) were performed manually (non-automated) in quadruplicates, at RT and using a total reaction volume of 50 μL in 96-well plates. The colorimetric reaction was initiated by adding 200 μL BIOMOL GREEN reagent and after 20 min incubation at RT the A_650_ was measured with a MultiScan EX plate reader (Thermo SCIENTIFIC). The interference of reagents (DMSO, NaCl, glycerol, DTT, SP and GSH) with the colorimetric reaction was evaluated individually using 20 μM K_2_HPO_4_ as Pi standard. The assay conditions (e.g. reagents, TryS units, time) were adjusted to yield optimal signal detection (20 μM Pi) taking into account the intracellular concentration reported or estimated for each substrate and/or to avoid interference with the colorimetric reaction.

The linearity range, detection limit (e.g. [Pi] corresponding to 3σ^n-1^ the A_650nm_ of the blank) and interference was estimated from plots of blank-corrected A_650nm_ *vs*. [Pi] or the reagent concentration for each condition tested. The plots were prepared and analyzed with OriginPro 8 (OriginLab Corporation). The errors are expressed as 2σ^n-1^.

#### Screening assay

For all TryS, ATP was used at 150 μM because of high background signal at higher concentrations and SP fixed at 2 mM, in agreement with the intracellular levels calculated from data reported in the literature for trypanosomatids [58–60]. GSH was adjusted to 0.05, 0.57 and 0.25 mM for *Tb*TryS, *Tc*TryS and *Li*TryS, respectively, to avoid substrate inhibition or to approach physiological concentrations [14].

A master mix (MM) solution containing all the substrates at 1.25-fold their end concentration in assay was prepared in screening reaction buffer (5 mM DTT, 10 mM MgSO_4_, 0.5 mM EDTA, 100 mM HEPES pH 7.4, 9 mM NaCl, 10% v/v DMSO) and kept on ice until use. Microtiter plate wells were loaded with 5 μL of test compound, DMSO (reaction control) or TryS-specific inhibitor (inhibition control) and 40 μL of MM. The reactions were then started by adding 5 μL of TryS (1, 2 and 3 x 10^−5^ μmol.min^-1^.mL^-1^ for *Tc*TryS, *Li*TryS and *Tb*TryS, respectively) and stopped after 15 min with 200 μL BIOMOL GREEN reagent.

The compounds used as inhibition control were added at concentrations that inhibited 50% TryS activity: 30 μM and 150 nM MOL2008 for *Tb*TryS and *Li*TryS, respectively, and 30 μM J18 for *Tc*TryS. Blanks were prepared for each condition by adding 5 μL of screening reaction buffer with 150 mM NaCl instead of enzyme.

The TryS activity is calculated as follow: % TryS activity = {[(*A*_*650nm Ci*_−*A*_*650 nm CiB*_) / (*A*_*650nm E*_−*A*_*650nm EB*_)] x 100}, where *A*_*650 nm Ci*_ is the mean absorbance at 650 nm for the reaction test with compound i, *A*_*650 nm CiB*_ is the mean absorbance at 650 nm for the blank control with compound i, *A*_*650 nm E*_ is the mean absorbance at A_650 nm_ for the reaction control with DMSO and *A*_*650 nm EB*_ is the mean absorbance at A_650 nm_ for the blank control with DMSO. The assays yielded an intra-assay coefficient variation (*CV = σ*^*n-1*^_*E*_ / E) ≤ 2.5%, a signal to background coefficient (E/E_B_) of ~ 3.5 and a Z’ factor [61] ≥ 0.85.

#### Screening work-flow

The solubility in DMSO of each compound was evaluated immediately prior to assay. Compounds insoluble at 3 mM in DMSO were excluded from the screening. Soluble compounds were stepwise diluted in DMSO up to 0.3 mM. For the primary screening all compounds were tested (inhibition and blank) at a fixed concentration of 30 μM. Compounds that decrease or increase TryS activity ≤ 55% or ≥ 105%, respectively, were tested for interference as described in S1 Text.

For compounds that at 30 μM decreased TryS activity ≤ 55%, the IC_50_ values were obtained from 10-point dose-response curves (% TryS activity ± 2σ^n-1^ *vs*. Log nM compound) fitted to a four-parameter sigmoidal equation (Boltzmann model). The reaction conditions were identical to those described for the screening assay and, whenever corresponding, the TryS activity values were corrected by the interference at each compound’s concentration.

### Biology

#### Proliferation assays for parasites and macrophages

The biological activity of selected compounds was evaluated in the infective form of *T*. *b*. *brucei* strain 427, cell line 514–1313 (WT) and cell line RNAi-TryS [16], as well as *Leishmania infantum* promastigotes (MHOM MA67ITMAP263; kindly provided by Dr. Helena Castro and Dr. Ana Tomas, IBMC, Portugal). The assay conditions to assess the cytotoxicity of compounds towards parasites and murine macrophages (cell line J774) are described in S1 Text. All assays were performed in duplicates or triplicates.

#### Western blot analysis of TryS expression

Total cell extracts from the WT and RNAi-TryS cell lines of *T*. *b*. *brucei* grown in the presence or absence of oxytetracycline were separated under reducing conditions on an SDS / 10% PAGE. The blotted membranes were incubated with mouse polyclonal antibodies against *Tb*TryS (dilution 1:1000) followed by a horseradish peroxidase-conjugated anti-mouse IgG (dilution 1:10000). Reactive bands were detected by chemiluminescence using ECL plus (GE Healthcare) and quantified by densitometric analysis using the free software ImageJ (http://imagej.nih.gov/ij/).

#### Analysis of thiols

The method is a modified version of that described in [62]. Briefly, *L*. *infantum* promastigotes or bloodstream *T*. *b*. *brucei* (WT and RNAi-TryS cell lines) treated or not with compounds (MOL2008 or EAP1-47, respectively) for 24 h were harvested by centrifugation (2000*g*, 10 min at 4°C), washed twice with PBS and resuspended at a density of 10^8^ cells/0.1 mL of 40 mM Hepes pH 8.0 and 2 mM mono-bromobimane in methanol. After incubation at 70°C for 3 minutes, the samples were cooled on ice, then added of 100 μL 4 M methanesulfonic acid (adjusted to pH 1.5 with LiOH) and incubated overnight at 4°C. The supernatant containing the derivatized low molecular mass thiols was centrifuged at 13000*g* 40 min at 0°C. Labeled thiols were separated using solvent A: 0.25% camphorsulfonic acid pH 2.64 and solvent B: 25% 1-propanol in solvent A and the following conditions: 100 μL of the sample, corresponding to 5x10^7^ cells, was injected onto a C18, 5 μm, 4.6 x 150 mm column connected to an HPLC (both from Agilent) pre-equilibrated in 12% solvent B followed by a linear gradient from 12–50% solvent B over 36 minutes at a flow of 1 mL/min. Specific thiol concentrations were determined from peak areas relative to different masses of derivatized standards (i.e. 100, 75, 50, 25, 10, 5 and 0.5 nmol) of GSH and T(SH)_2_, the last produced according to [63].

### Accesion numbers

AAO00722.1; CAC87573.1; CAM69145.1

## Results

### Kinetic parameters of TryS and its implicancies for enzyme regulation and inhibition

The kinetic studies of TryS employed in this work were performed with three major aims, first to test the quality of the recombinant enzymes and establish optimal assay conditions, second, to disclose kinetic data previously not addressed for TryS from *T*. *cruzi* strain Tulahuen 0 and *L*. *infantum* (strain JPCM5) and a reaction product (ADP), and third, to compare the kinetic behavior of different TryS and its implication for enzyme regulation and inhibition. The kinetic data available for TryS from earlier [18; 22–27] and the present work are presented in Table 1 and S1 Fig.

**Table 1:** Kinetic parameters for recombinant tritryp trypanothione synthetase (TryS).

Parasite	*T*. *cruzi*			*T*. *b*. *brucei*				*L*. *major* [26]	*L*. *infantum* [20]	*C*. *fasciculate* [23]
Strain	Silvio X10 clone 7 [25]	Tulahuen 0	Ninoa [22]	427 (MITat1.4) [27]			427^a^ [24]	Friedlin	JPCM5	US^b^
K_M_ GSH^c^	570 ± 50	**140 ± 13**	760 ± 210	56.2 ± 10.7^d^	23.8 ± 2.3^e^	**142 ± 20**	34 ± 4	89 ± 7	**170 ± 23**	1175
K_i_ GSH	1200 ± 100	**1690 ± 172**	No^f^	36.5 ± 6.7^d^	55 ± 6^e^	**254 ± 45**	143 ± 20	1000 ± 80	**699 ± 75**	Yes
K_M_ SP^g^	625 ± 39	**702 ± 70**	860 ± 95	37.8 ± 5.0^d^	45.4 ± 2.0^e^	**238 ± 25**	92 ± 25	940 ± 140	**1434 ± 135**	7424
K_M_ ATP	53 ± 3	**41 ± 6**	70 ± 40	7.1 ± 0.4^d^	8.6 ± 0.6^e^	**19 ± 5**	6.6 ± 0.5	63 ± 2	**43 ± 7**	52
K_i_ ADP	ND^h^	**60 ± 6**	ND^h^	ND^h^	ND^h^	**40 ± 5**	ND^h^	ND^h^	**90 ± 8**	ND^h^

The apparent *K*_*M*_ and *K*_*i*_ values (μM) were determined using the lactate dehydrogenase (LDH)/pyruvate kinase (PK) assay and the *K*_*i*_ ADP was assessed using the end-point assay. Whenever available, the values are expressed as the mean ± S.D.. The parameters determined for the N-terminal His-tagged tritryp TryS studied in this work are shown in bold letters and the references to data published earlier in brackets. Please, refer to S8 Table for details about the conditions employed in the kinetic characterization of each enzyme.

^a^ *Trypanosoma brucei brucei*, strain 427, cell line 449.

^b^ US, unknown strain.

^c^ GSH, glutathione

^d^ Kinetic parameters determined for untagged *Trypanosoma brucei* TryS (*Tb*TryS) using the PK/LDH coupled assay [27].

^e^ Kinetic parameters determined for untagged *Tb*TryS using the end-point assay [18].

^f^ No, denotes lack of GSH inhibition at 4.5 mM.

^g^ SP, spermidine.

^h^ ND, not determined.

*Li*TryS presented apparent *K*_*M*_ values of 166 ± 62 μM for GSH, 1335 ± 167 μM for SP and 42 ± 10 μM for ATP, and a apparent *K*_*i*_ for GSH of 680 ± 160 μM, all of which are in the same order of magnitude as those reported for non-tagged TryS from the related species *L*. *major* [26].

The apparent kinetic parameters of *Tc*TryS strain Tulahuen 0 (*K*_*M*_ GSH = 123 ± 23 μM, *K*_*M*_ SP = 685 ± 105 μM, *K*_*M*_ ATP = 41 ± 6 μM and *K*_*i*_ GSH = 1600 ± 230 μM) are similar to those published for *Tc*TryS strain Silvio X10 clone 7 [25] and strain Ninoa [22], except that inhibition by GSH was not observed for TryS from the last strain [22].

The apparent *K*_*M*_ values for substrates and *K*_*i*_ for GSH obtained for His-tagged *Tb*TryS (*K*_*M*_ GSH = 135 ± 43 μM, *K*_*M*_ SP = 238 ± 51 μM, *K*_*M*_ ATP = 18 ± 6 μM and *K*_*i*_ GSH = 242 ± 102 μM) were almost 3- to 7-fold higher than those reported for the untagged version of this protein (*K*_*M*_ GSH = 23.8–56.2 μM, *K*_*M*_ SP = 37.8–92 μM, *K*_*M*_ ATP = 6.6–8.6 μM and *K*_*i*_ GSH = 36.5–143 μM) by different laboratories [24, 27], which may be ascribed to different assay conditions (summarized in S8 Table) as highlighted in previous publications [23, 24]. Nonetheless, all values determined here for His-tagged tritryp TryS differed in less than one order of magnitude from those reported for tag-free versions from identical or homologue proteins, hence the recombinant form of the enzymes were rated as suitable for the screening assay.

Inhibition by ADP has been reported to occur for *Cf*GspS with a *K*_*i*_ of 80 μM [64] but information is lacking for related enzymes from pathogenic trypanosomatids. As shown here, ADP competed for the ATP-binding site of *Tb*TryS, *Li*TryS and *Tc*TryS (S1 Fig) with apparent *K*_*i*_ values of 40 ± 5 μM, 90 ± 8 μM and 60 ± 6 μM, respectively (Table 1). It is worth to note that these *K*_*i*_ values represent overall estimates of product inhibititon because the assay conditions used do not allow distinguishing a preferential inhibition of the first or second biosynthetic step catalyzed by TryS.

Comparison of the kinetic parameters for TryS from three major trypanosomatid species obtained under similar experimental conditions (this study), shows that the enzyme from African trypanosomes presents *K*_*M*_ values for ATP and SP that are remarkable lower (2.3‒5.6-fold) than those for TryS from *L*. *infantum* and *T*. *cruzi* (Table 1). A similar conclusion can be drawn from studies carried out for related TryS in other laboratories [18, 22, 24–27]. At variance with *Leishmania* spp. and *T*. *cruzi*, *T*. *brucei spp*. is an extracellular pathogen that fully relies on *de novo* synthesis of polyamines and glycolysis to fulfil its metabolic and energetic needs [58, 65]. Thus, TryS from African trypanosomes presents kinetic parameters that guarantee the production of the indispensable metabolite trypanothione [16, 17] under conditions of restricted supply of ATP and SP that the parasite may face during its complex life cycle (e.g. differentiation, which entails drastic reprogramming of energetic metabolism, and different nutrient availability in vector and host). All three tritryp TryS displayed similar *K*_*M*_ values for GSH, which contrast with earlier studies reporting a higher affinity of *T*. *b*. *brucei* TryS for this substrate [18, 22, 24, 27]. As previously reported [18, 23–27], tritryp TryS were susceptible to substrate inhibition by GSH, although the inhibitory efficiency varied between species as follows: *T*. *b*. *brucei* (apparent *K*_*i*_/*K*_*M*_ GSH = 1.8) > *L*. *infantum* (apparent *K*_*i*_/*K*_*M*_ GSH = 4.1) > *T*. *cruzi* (apparent *K*_*i*_/*K*_*M*_ GSH = 13.0). This suggests that the *T*. *b*. *brucei* enzyme is particularly sensititive to inhibition by the substrate GSH. However, the physiological role of this inhibition mechanism, if any, remains questionable because substrate accumulation will further enhance TryS inhibition and GSH cannot surrogate T(SH)_2_ functions in *T*. *brucei* [16]. For the leishmanial and *T*. *cruzi* TryS, substrate inhibition will become relevant only at high concentrations of GSH (e.g. > 1.5 mM), which, except for a few examples of *L*. *donovani* parasites grown to mid-log phase (e.g. 2.27 mM GSH for axenic amastigotes from the strain BOB and 1.68 mM for promastigotes from the strain LV9) [14], appears to be a non-physiological condition. Indeed, *L*. *infantum* parasites harbouring a single *trys* allele and with a TryS content about 50% lower than that of wildtype cells show no phenotype *in vitro* and in infected animals [20].

The *K*_*i*_ ADP / *K*_*M*_ ATP ratios for tri-tryp TryS range from1.5 to 2.2-fold, suggesting that ADP may be a physiological modulator of TryS activity. However, taking into account that the intracellular concentration of ATP for trypanosomatids (e.g. 2–4 mM and 0.58 mM for infective *T*. *b*. *brucei* [65, 66] and *T*. *cruzi* [67], respectively, and 0.87 mM for *L*. *donovani* promastigotes [68]) is > 14-fold in excess with respect to the respective *K*_*M*_ values for tritryp TryS (e.g. 18–60 μM; Table 1) and that the ATP/ADP ratio reported or estimated by us for trypanosomatids is between 3 to 10 [65, 66, 69–71], such regulatory role of ADP in T(SH)_2_ biosynthesis may be questioned. A recent kinetic analysis of *Tb*TryS assisted by computational modeling of the enzymatic mechanism [24] highlighted that the activated enzyme is particularly sensitive to inhibition by GSH and T(SH)_2_. Because the experimental setup employed in this study did not consider inhibition by ADP and excluded ADP as an integral component of the enzyme activated complex, the real contribution of this product to TryS inhibition remains to be addressed.

### Screening

#### Assay development and validation

Under reaction conditions resembling the screening assay and using KH_2_PO_4_ as Pi standard, the detection limit and linearity range of the colorimetric reaction with BIOMOL GREEN was ~ 1.9 μM Pi and 2‒50 μM Pi, respectively (S2 Fig). DTT and NaCl (salt present in enzyme buffer) added up to 5 mM and 30 mM, respectively, do not affect assay’s readout (S3A and S3D Fig). DMSO (compound solvent) and glycerol (cryoprotective agent added to TryS) interfered with BIOMOL GREEN signal in an additive fashion. For instance, 10% v/v DMSO (e.g. concentration used in the assay) and 4% v/v glycerol reduced assay sensitivity 8.4 ± 1.5% (n = 8) and ~ 12% (S3C Fig), respectively, whereas addition of both reagents (DMSO 10% v/v and glycerol 4% v/v) decreased ~ 25% assay sensitivity (S3D Fig). In order to reduce the signal interference, assays were performed at glycerol concentrations < 1% v/v.

Assay linearity is affected at ATP concentrations > 150 μM due to increase background signal and ADP-mediated inhibition of TryS (S1 Fig; see previous section). Thus, for practical reasons ATP was adjusted to 150 μM, a concentration that is 4‒27-fold lower than the estimated physiological concentration of the substrate in different trypanosomatids [66–70] but ~3.5 and 8-fold higher than the *K*_*M*_ values for *Tc*TryS and *Li*TryS, and *Tb*TryS, respectively (Table 1).

GSH added up to 2.5 mM does not interfere with the colorimetric signal. For assays with *Tc*TryS, GSH was used at 570 μM, a value within the physiological concentrations reported for infective forms of *T*. *cruzi* strains (i.e. 340 to 1180 μM) [14]. For the screening involving *Li*TryS, GSH was employed at a concentration of 250 μM, which is near the intracellular content estimated for amastigote forms of *L*. *donovani* (250 μM) and *L*. *major* (310 μM) isolated from hamsters and mice, respectively [14]. In order to avoid substrate inhibition of *Tb*TryS, GSH was adjusted to 50 μM a value close to the corresponding *K*_*M*_ (Table 1) and 5- fold lower than the intracellular concentration (i.e. 230–260 μM) reported for this substrate in trypanosomes isolated from infected rats [14], respectively.

Irrespective of the TryS species, SP, a substrate not interfering with the colorimetric reagent (S3B Fig), was fixed to 2 mM for the screening assays. This concentration is 8.4-, 2.9- and 1.5-fold higher than the *K*_*M*_ values for *Tb*TryS, *Tc*TryS and *Li*TryS, respectively and close to that we estimated for the clinically relevant forms of *T*. *b*. *brucei* (SP ~ 2 mM for bloodstream stage), *T*. *cruzi* (~ 3 mM for amastigotes and trypomastigotes) and *L*. *mexicana* (~ 7 mM for amastigotes) according to information available on polyamine content [58–60], cell volumes and/or total protein content per cell for each parasite species [14, 71, 72].

The linearity A_650nm corr_ *vs*. time was evaluated for each TryS at substrates concentrations used in the screening assay (see Experimental Procedures, S4 Fig). For *Li*TryS (2.3 x 10^−5^ μmol.min^-1^.mL^-1^), the assay was linear for at least 30 min whereas the production of Pi by *Tc*TryS (3.5 x 10^−6^ μmol.min^-1^.mL^-1^) and *Tb*TryS (1.5 x 10^−5^ μmol.min^-1^.mL^-1^) displayed a linear relation up to 15 min. As indicated above, the trypanosomal TryS were more sensitive than *Li*TryS to inhibition by ADP, which may explain the lower turnover of the former at longer reaction times. Therefore, a reaction end-point of 15 min was selected for the screening assays, which corresponds to the formation of 20 μM Pi and ADP for all three enzymes.

The assay conditions reported above for each enzyme yielded a Z’ factor [61] of ≥0.85, a signal background coefficient (E/EB) of ~ 3.5 and a CV (σ^n-1^_E_/E) intra-assay ≤ 2.5%, which validate the screening test as highly robust and reproducible.

#### Compound screening at near physiological concentrations of substrates

**Fig 2:**
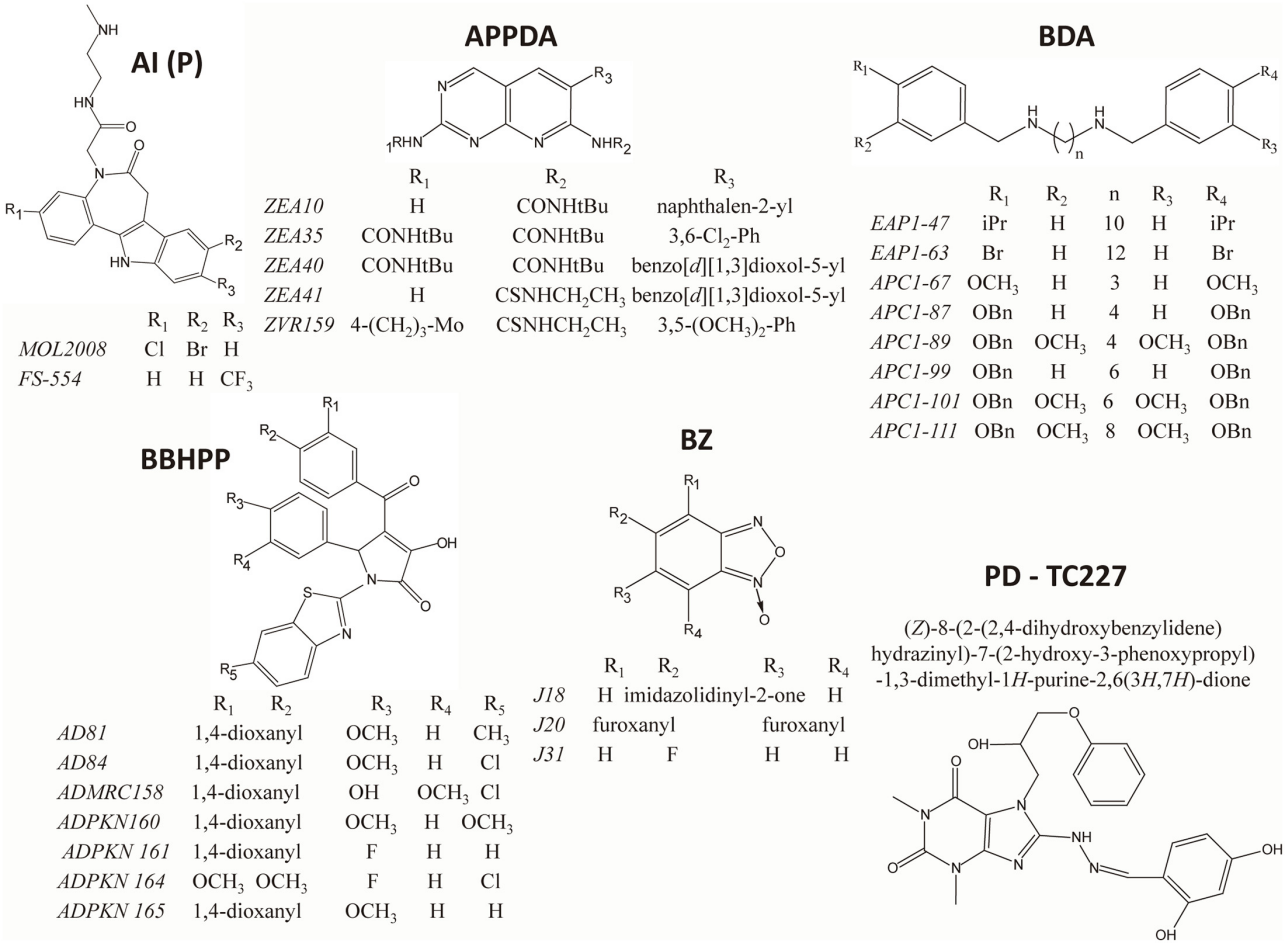
Structure of compounds affecting tritryp trypanothione synthetase activity. **AI (P)**, 4,5-dihydroazepino[4,5-*b*]indol-2(1*H*,3*H*,6*H*)-one derivatives, paullones derivatives, (FS-554 and MOL2008), five APPDA, 6-arylpyrido[2,3-*d*]pyrimidine-2,7-diamine derivatives (ZEA10, ZEA35, ZEA40, ZEA41 and ZVR159), eight BDA, *N*,*N'*-bis(3,4-substituted-benzyl) diamine derivatives (EAP1-47, EAP1-63, APC1-67, APC1-87, APC1-89, APC1-99, APC1-101 and APC1-111), seven BBHPP, 1-(benzo[*d*]thiazol-2-yl)-4-benzoyl-3-hydroxy-5-phenyl-1*H*-pyrrol-2(5*H*)-one derivatives (AD81, AD84, ADMRC158, ADKPN160, ADKPN161, ADKPN164 and ADKPN165), three BZ, benzofuroxan derivatives (J18, J20 and J31) and one PD, 1*H*-purine-2,6(3*H*,7*H*)-dione derivatives [(*Z*)-8-(2-(2,4-dihydroxybenzylidene)hydrazinyl)-7-(2-hydroxy-3-phenoxy propyl)-1,3-dimethyl-1*H*-purine-2,6(3*H*,7*H*)-dione, TC227]. iPr, tBu, OBn, Mo and Ph, correspond to an isopropyl, tert-butyl, O-benzyl, 4 -morpholinyl and phenyl substitution, respectively.

The screening work flow is shown in Fig 2 and all data here reported correspond to assays that fulfilled the quality control parameters indicated above.

**Fig 3:**
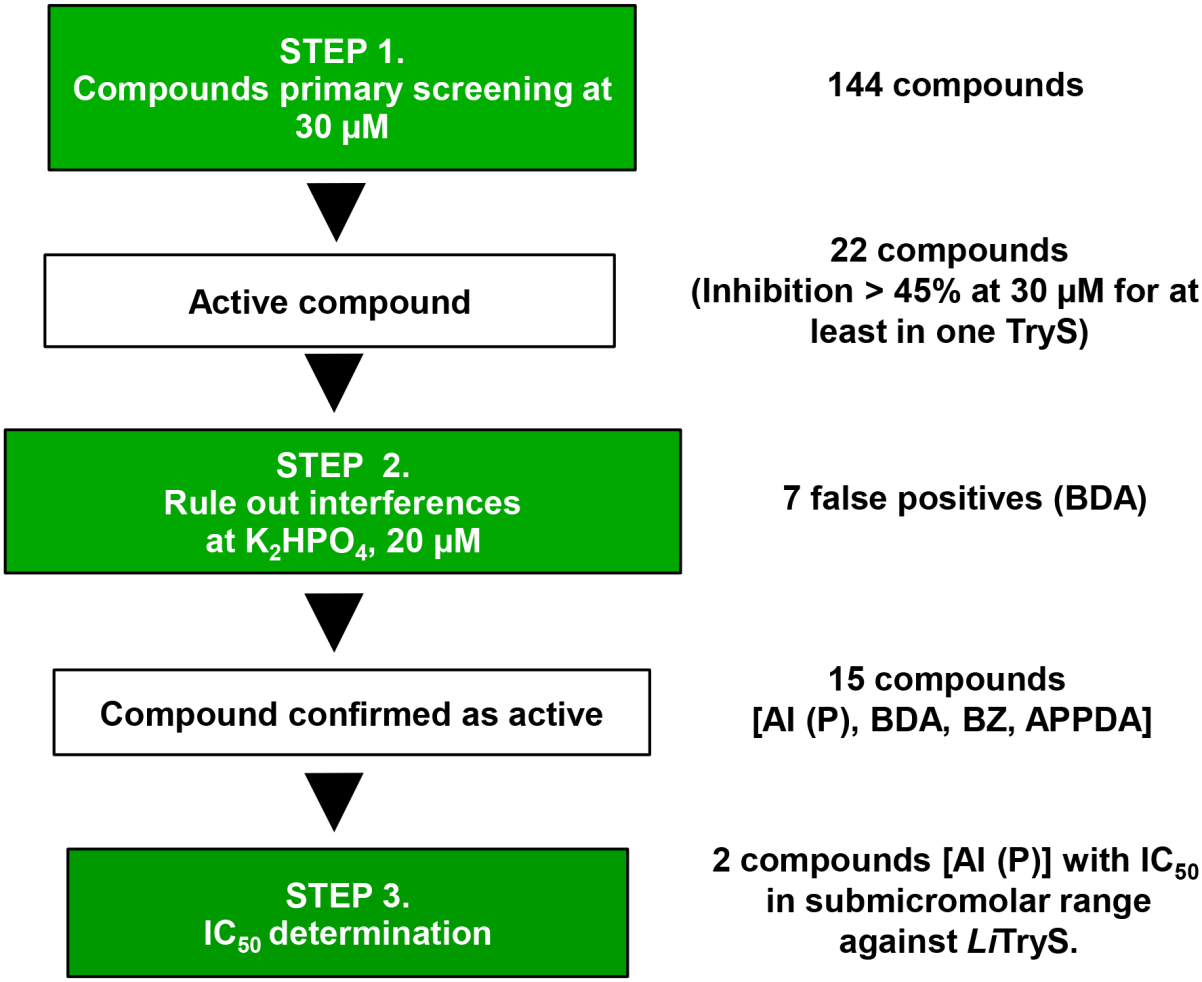
Screening work flow. The different steps, the most relevant assay conditions and the go/no-go criteria of the screening campaign are indicated in boxes. The figures on the right refer to the number of compounds screened and that subsequently advanced during the campaign. From 144 compounds, 22 compounds lowered assay signal ≥ 45% for at least one TryS. From these 22, 7 BDA were false positive and the remaining 15 compounds were confirmed as enzyme inhibitors. Two of them are **AI** with potency in the submicromolar range against *Li*TryS. AI (P), 4,5-dihydroazepino[4,5-*b*]indol-2(1*H*,3*H*,6*H*)-one derivatives (P, paullone); APPDA, 6-arylpyrido[2,3-*d*]pyrimidine-2,7-diamine derivatives; BZ, benzofuroxan derivatives; BDA, *N*,*N'*-bis(3,4-substituted-benzyl) diamine derivatives.

The initial screening performed with 144 compounds belonging to 7 different families (Fig 3 and S1–S7 Tables) and tested at 30 μM against tritryp TryS, allowed the identification of 22 compounds that lowered Pi signal to ≤ 55%. An assay interference counter-screen confirmed 15 of them as true TryS inhibitors (Table 2) and the remaining 7, all being **BDA**, as false positive readouts (S3 Table).

**Table 2:** Inhibitors of tritryp trypanothione synthetase (TryS) identified in this work and their biological activity.

Compound	Structure^a^	*Tc*TryS	*Li*TryS	*Tb*TryS	*T*. *b*. *Brucei* EC_50_ (μM)^b^	*L*. *infantum* EC_50_ (μM)^c^	SI^d^
MOL2008	AI (P)	40.5 ± 5.9%; (1.00)	0.15 ± 0.06 μM ^e^; (0.99); 0.35 ± 0.03 ^f^	59.0 ± 6.0%; (0.97)	4.3 ± 0.7	12.6 ± 1.6	2.4 (0.8)
FS-554	AI (P)	55.5 ± 3.8%; (1.02)	0.35 ± 0.05 μM ^e^; (0.99); 0.36 ± 0.04 ^f^	~75 μM; (0.94 at 300 μM)	ND	112.3 ± 1.1 ^g^	ND (0.6)
ZEA10	APPDA	32.3 ± 4.0%	32.1 ± 6.4%	48.7 ± 2.8%; (1.41)	0.28 ± 0.08	ND	>4
ZEA35	APPDA	61.3 ± 7.6%; (1.01)	13.8 ± 9.7%	24.3 ± 6.1%	ND	ND	ND
ZEA40	APPDA	52.2 ± 1.4%; (1.02)	15.3 ± 8.3%	25.3 ± 5.2%	ND	ND	ND
ZEA41	APPDA	65.4 ± 1.5%; (0.99)	13.6 ± 3.4%	20.9 ± 4.4%	ND	ND	ND
EAP1-47	BDA	53.5 ± 1.3%; (1.48)	47.8 ± 1.8%; (1.34)	51.1 ± 4.2%; (1.49)	0.20 ± 0.02	ND	15
EAP1-63	BDA	30.8 ± 3.8%	25.9 ± 9.3%	47.5 ± 2.8%; (1.30)	0.090 ± 0.007	ND	124
EAP1-67	BDA	23.8 ± 6.6%; (1.34)	51.5 ± 5.3%; (1.35)	42.4 ± 1.6%; (1.33)	ND	ND	ND
APC1-87	BDA	26.6 ± 4.1%	56.7 ± 7.9%; (1.26)	37.9 ± 4.0%	ND	ND	ND
APC1-89	BDA	25.6 ± 1.4%	23.4 ± 2.7%; (1.32)	50.9 ± 1.5%; (1.31)	0.061 ± 0.001	ND	164
APC1-99	BDA	33.4 ± 4.1%	39.1 ± 3.4%	48.9 ± 1.7%; (1.05)	0.015 ± 0.001	ND	67
APC1-101	BDA	17.0 ± 2.1%; (1.40)	26.3 ± 10.1%; (1.36)	61.2 ± 1.8%; (1.33)	0.28 ± 0.09	ND	10
APC1-111	BDA	28.9 ± 4.1%; (1.57)	49.6 ± 5.7%; (1.51)	55.6 ± 3.2%; (1.52)	0.040 ± 0.001	ND	67
J18	BZ	63.5 ± 1.5%; (1.00)	41.3 ± 3.6%; (1.00)	7.3 ± 5.9%	ND	ND	ND

*In vitro* and *in vivo* (cell) activity profile of most promising TryS inhibitors. Enzyme inhibition is expressed as % TryS inhibition ± 2σ^n-1^ and IC_50_ values in μM concentration ± 2σ^n-1^. For compounds affecting BIOMOL GREEN signal, the interference factor used to correct TryS activity is provided in brackets (see S1 Text). All values reported stem from at least duplicates. ND, not determined.

^a^ the chemical scaffolds are: AI (P), 4,5-dihydroazepino[4,5-*b*]indol-2(1*H*,3*H*,6*H*)-one derivatives, paullone derivatives; APPDA, 6-arylpyrido[2,3-*d*]pyrimidine-2,7-diamine derivatives; BDA, *N*,*N'*-bis(3,4-substituted-benzyl) diamine derivatives; BZ, benzofuroxan derivatives.

^b^ Cytotoxicity against bloodstream *Trypanosoma brucei brucei* expressed as effective concentration 50: EC_50_.

^c^ Cytotoxicity against *Leishmania infantum* promastigotes is expressed as effective concentration 50: EC_50_.

^d^ Selectivity index calculated as the ratio: EC_50_ for murine macrophages (cell line J774) *vs*. EC_50_ for *T*. *b*. *brucei* or EC_50_ for *L*. *infantum* (value in brackets).

^e^ IC_50_ value calculated from 3 independent experiments and expressed as mean ± 2 S.D.

^f^ Slope from IC_50_ plots calculated from 3 independent experiments and expressed as mean ± 2 S.D.

^g^ EC_50_ value reported by [20]

**Fig 4:**
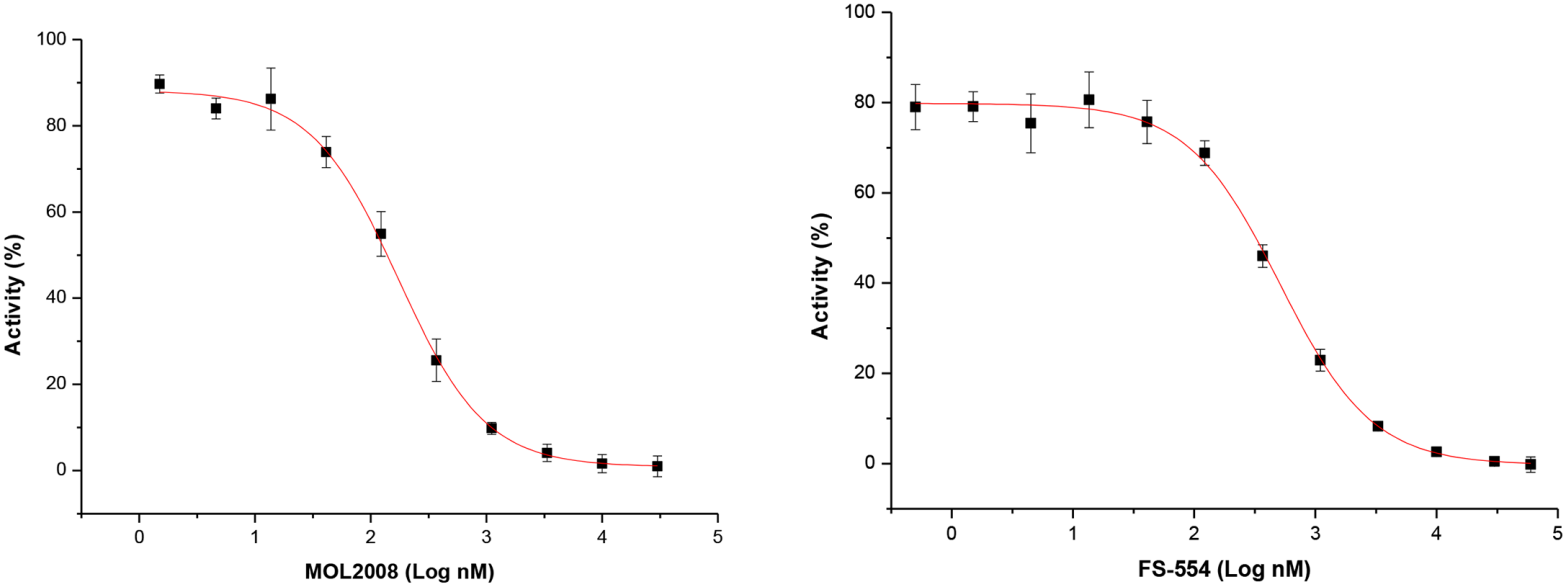
Inhibition plot of *Leishmania infantum* trypanothione synthetase (TryS) by paullones. The data are presented as mean TryS activity (%) ± 2 S.D. (n = 4) as function of Log_10_ concentration (nM) of compound adjusted to a four-parameter Boltzmann sigmoidal equation. Representative dose-response plot obtained for MOL2008 (IC_50_ = 0.14 ± 0.03 μM; slope plot 0.38 ± 0.03) and for FS-554 (IC_50_ = 0.32 ± 0.73 μM; slope plot 0.37 ± 0.03).

There was no correlation between the rate of hit identification for each enzyme (i.e. 5.6% for *Tb*TryS and 4.2% for *Tc*TryS and *Li*TryS), and the potency of the compounds (i.e. 2 out 6 were nM inhibitors of *Li*TryS whereas all compounds targeting the trypanosomal TryS were two digit μM inhibitors, see Table 2). Supporting the existence of species-specific differences between TryS, the individual enzymes were preferentially targeted: *Li*TryS by **AI**; *Tb*TryS by **BDA** and *Tc*TryS by **APPDA**. Only 4 compounds out of 15, each two **BDA** and **AI**, were able to target multiple TryS, though to different extent. EAP1-47 [*N*^*1*^,*N*^*10*^-bis(4-isopropylbenzyl)decane-1,10-diamine] and APC1-111 [*N*^*1*^,*N*^*8*^-bis(4-(benzyloxy)-3-methoxybenzyl)octane-1,8-diamine] at 30 μM halved activity of all three TryS or *Tb*TryS and *Li*TryS, respectively; MOL2008 and FS-554 were two digits nM inhibitors of leishmanial TryS (Fig 4) that, to a remarkable minor degree (~ 50% inhibition at 30 μM), also targeted both trypanosomal enzymes or *Tc*TryS, respectively.

Early investigations had shown that the *N*^*5*^-substituted paullone derivatives MOL2008 and FS-554 are potent inhibitors of TryS from *C*. *fasciculata* (IC_50_ = 30 nM) [10] and *L*. *infantum* (IC_50_ = 350 nM) [20], respectively, but information on their activity towards the homologue enzyme from related trypanosomatids was missing. MOL2008 was the most potent inhibitor of *Li*TryS (IC_50_ = 0.15 ± 0.06 μM) identified in the screening that displayed a moderate activity against *Tb*TryS (IC_50_ ~ 30 μM) and *Tc*TryS (40% TryS inhibition at 30 μM; see Table 2). On the other hand, FS-554 showed moderate activity against *Tc*TryS (~50% inhibition at 30 μM) and a weak activity against *Tb*TryS (~50% inhibition at 75 μM) (Table 2). Substitution of position *N*^5^ appears key to confer anti-*Li*TryS activity to **AI** since the inhibitory activity of unsubstituted derivatives was almost abrogated (compound 11a and 11b in S5 Table). Given the amine nature of the *N*^*5*^ substitutent, a potential SP-competitive mechanism of MOL2008 on *Li*TryS was investigated. Assays performed at fixed concentration of co-substrates (250 μM GSH and 150 μM ATP) and variable concentrations of SP (0.2 to 16 mM) show that enzyme inhibition was inversely proportional to polyamine concentration (S9 Table) and confirmed the proposed inhibition mechanism. A collection of 4-azapaullones substituted in position 9 and 11 was recently assayed for their anti-*T*. *b*. *brucei* and *Tb*TryS activity [56]. Only derivatives containing in position 9 an α,β-unsaturated carbonyl substituted with a phenyl, phenyl(amino) or benzylamino group showed a minor anti-*Tb*TryS activity (30–40% inhibition at 30 μM). Given the species-specificity displayed by tri-tryp TryS, the complete set of 4-azapaullones was tested against the *L*. *infantum* and *T*. *cruzi* enzyme (S5 Table). None of the compounds inhibited *Tc*TryS to a significant level (enzyme inhibition ≤ 17% at 30 μM compound) whereas *Li*TryS showed again to be more susceptible to inhibition by the paullone scaffold, with 4-chloro-phenyl > furan-2-yl > 4-methoxy-phenyl analogues in 9-position reducing 32–44% enzyme activity at 30 μM. Identical substitutions (i.e. 4-methoxy-phenyl and 4-chloro-phenyl) in 11-position of 4-azapaullones rendered analogues with similar inhibitory activity (30–35%) towards *Li*TryS.

The **BDA** (S5 Fig, S3 Table) exerted a low to moderate inhibition of TryS that allows performing a SAR analysis. For all TryS, the inclusion of bulky substitutions (R^1^ = R^4^ = OBn: APC1-87, APC1-99 and APC1-109, or in addition R^2^ = R^3^ = OMe: APC1-111, APC1-101 and APC1-89) at the benzyl rings yields derivatives with higher activity (40–60% inhibition) than the unsubstituted congeners and analogues containing less bulky groups (R^2^ = R^3^ = OMe, R^1^ = R^4^ = OH) (S5A Fig). In contrast to *Li*TryS, the trypanosomal enzymes were particularly sensitive to the length of the linker, with derivatives bridged by 3- or 10-carbon being less active. This suggests a substituent additive effect to the length of the carbon chain with a possible steric fulfillment demand of the interacting pocket. For the halogenated **BDA**, the length of the linker appears to be a common determinant of activity against all TryS, whereas each enzyme displayed certain specificity for the nature of the halogen atom (S5B Fig). Thus, derivatives with a 12-carbon linker are more potent TryS inhibitors than those consisting of shorter chains and the halogen substitution yielding the highest activity (~50% inhibition at 30 μM) for this scaffold is F (EAP1-69), Cl (EAP1-67) and Br (EAP1-63) for *Tc*TryS, *Li*TryS and *Tb*TryS, respectively. On the other hand, the inclusion of an isopropyl group at position R_1_ = R_4_ of **BDA** with a 10-carbon linker (EAP1-47) improved up to 40% tri-tryp TryS inhibition when compared to the unmodified compound EAP1-9 and produced the best multi-TryS inhibitor here identified (50% inhibition at 30 μM).

From a group of 35 **APPDA** tested, three derivatives containing a substituted phenyl or benzyl group in position R3 (i.e. ZEA35, ZEA 40 and ZEA 41) showed selectivity and a moderate potency towards *Tc*TryS (50% inhibition at 30 μM; Table 3). Replacing the R3 benzyl of ZEA41 by a naphthalen group (ZEA10) increased the activity against *Tb*TryS (50% inhibition at 30 μM) and *Li*TryS (17% increased inhibition) but was detrimental to *Tc*TryS inhibition (18% lower inhibitory activity). *Tc*TryS was also target of selective inhibition by several **BZ** with J18 being the most active (IC_50_ ~30 μM), and the derivatives J20 and J21 reducing about 40% enzyme activity when tested at 30 μM. J18 produced a similar degree of inhibition of *Li*TryS, whereas *Tb*TryS was almost refractory to inhibition by the **BZ** (S4 Table).

**Table 3:** Compounds enhancing tritryp trypanothione synthetase (TryS) activity.

Compound	Core structure^a^	*Tc*TryS	*Li*TryS	*Tb*TryS
ZVR159	APPDA	168.6 ± 4.1; (1.10)	100.6 ± 5.8	81.3 ± 1.6
AD81	BBHPP	109.7 ± 8.9	83.9 ± 4.2	128.2 ± 3.6; (0.93)
AD84	BBHPP	98.7 ± 5.1	82.1 ± 4.0	119.0 ± 6.3; (0.95)
ADMRC158	BBHPP	97.4 ± 6.1	85.3 ± 8.7	119.1 ± 4.6; (0.88)
ADPKN160	BBHPP	97.6 ± 3.7	77.9 ± 3.6	116.4 ± 4.2; (0.87)
ADPKN161	BBHPP	90.8 ± 5.8	83.5 ± 9.2	126.7 ± 0.8; (0.86)
ADPKN164	BBHPP	99.6 ± 5.7	66.0 ± 3.4	123.2 ± 7.0; (0.83)
ADPKN165	BBHPP	107.1 ± 4.5	83.5 ± 6.0	166.3 ± 5.5; (0.79)
J20	BZ	64.4 ± 3.8	111.5 ± 14.5; (0.97)	118.8 ± 2.4; (0.97)
J31	BZ	118.8 ± 6.1; (1.00)	84.1 ± 0.001	96.2 ± 3.7
TC227	PD	161.6 ± 5.3; (0.98)	88.5 ± 7.1	102.0 ± 2.3

Enzyme activity is expressed as % TryS activity ± 2σ^n-1^ and in brackets is provided the interference factor used to correct TryS activity of compounds affecting BIOMOL GREEN signal (see S1 Text). All values reported stem from at least triplicates. *Tc*TryS, *Tb*TryS and *Li*TryS are the trypanothione synthetase of *Trypanosoma cruzi*, *Trypanosoma brucei* and *Leishmania infantum*, respectively. ND, not determined.

^a^ the chemical scaffolds are: APPDA, 6-arylpyrido[2,3-*d*]pyrimidine-2,7-diamine derivative; BBHPP, 1-(benzo[*d*]thiazol-2-yl)-4-benzoyl-3-hydroxy-5-phenyl-1*H*-pyrrol-2(5*H*)-one derivatives; BZ, benzofuroxan derivatives; PD, 1*H*-purine-2,6(3*H*,7*H*)-dione derivative.

Certain **BBHPP** (ADMRC150, ADMRC154, ADMRC159 and ADPKN164) showed specificity for targeting *Li*TryS with an average inhibitory activity of 35% at 30 μM and marginal or, even, enhancing activity of trypanosomal TryS (Table 3 and S2 Table).

Under the assay conditions used for the screening, none of the **PD**, **AOCA** and singletons inhibited tri-tryp TryS to a significant level (S6 and S7 Tables). In this respect, prochlorperazine has been early reported as a SP-competitive inhibitor of *Tb*TryS with an IC_50_ of 19 μM, when assayed at a subsaturating concentration of the competing substrate (i.e. 25 μM SP) [18]. To investigate the potential of prochlorperazine to target TryS from other species and validate their mode of inhibition, we tested the activity of this compound against different TryS and at different SP concentrations. At 2 mM SP, the estimated intracellular concentration of this polyamine in trypanosomatids [58–60], none of the TryS was inhibited by prochlorperazine added at 30 and 300 μM. Lowering the SP concentration in the assay to 0.24 mM SP, 300 μM prochlorperazine reduces the activity of *Tb*TryS, *Tc*TryS and *Li*TryS by 58%, 34% and 0%, respectively. On the one hand, this result show that high intracellular concentrations of prochlorperazine have to be reached to impair T(SH)_2_ synthesis to a significant level and, on the other hand, highlights that more stringent assay conditions, as those representing nearly physiological substrate concentrations, will avoid the detection of hits with low pharmacological potential.

The screening also allowed the identification of compounds that increased 16–69% the activity of the trypanosomal TryS (i.e. for *Tb*TryS: 7 **BBHPP** and 2 **BZ** and for *Tc*TryS the **APPDA** ZVR159 and the **PD** TC227) (Table 3). Strikingly, none of the compounds tested here produced a similar stimulatory effect on the activity of the leishmanial enzyme. In order to shed light on the mechanism underlying the activation of TryS, the most active compounds were tested against trypanosomal TryS in the presence of different combinations of co-substrates. As shown in S10 Table, enhacement of TryS ATPase activity by non-chemically related compounds (ZVR159, TC227 and ADPKN165) was detected only when both co-substrates, GSH and SP, were present in the reaction mixture. These results are suggestive of an allosteric mechanism of TryS activation.

### Biological activity of compounds inhibiting TryS from African trypanosomes and *L*. *infantum*

The biological activity of the most active inhibitors of *Tb*TryS (i.e. IC_50_ ≤ 30 μM; MOL2008, ZEA10, EAP1-47, EAP1-63, APC1-89, APC1-99, APC1-101, APC1-111) and *Li*TryS (i.e. IC_50_ < 1 μM; MOL2008 and FS-554) was evaluated against the infective form of *T*. *b*. *brucei* or promastigotes of *L*. *infantum* and murine macrophages. Except for the paullone MOL2008 (EC_50_ 4.3 ± 0.7 μM), all other *Tb*TryS inhibitors presented anti-*T*. *b*. *brucei* activity in the nM range. The most active being the **BDA** APC1-99 (EC_50_ 15 ± 1 nM), APC1-111 (EC_50_ 40 ± 1 nM), APC1-89 (EC_50_ 61 ± 1 nM) and EAP1-63 (EC_50_ 90 ± 7 nM), followed by ZEA10, EAP1-47 and APC1-101 with EC_50_ between 200–280 nM (Table 2). In comparison to paullone FS-554 (EC_50_ 112.3 ± 1.1 μM) [20], MOL2008 displayed a 10-fold higher anti-leishmanial activity (EC_50_ 12.6 ± 1.6 μM).

The selectivity of the anti-parasitic effect was assessed using murine macrophages. Most compounds, except for MOL2008, FS-554 and ZEA 10, presented a selectivity index (SI) ≥ 10 with EAP1-63 (SI = 124) and APC1-89 (SI = 164) having the highest selectivity towards bloodstream *T*. *b*. *brucei* (Table 2).

**Fig 5:**
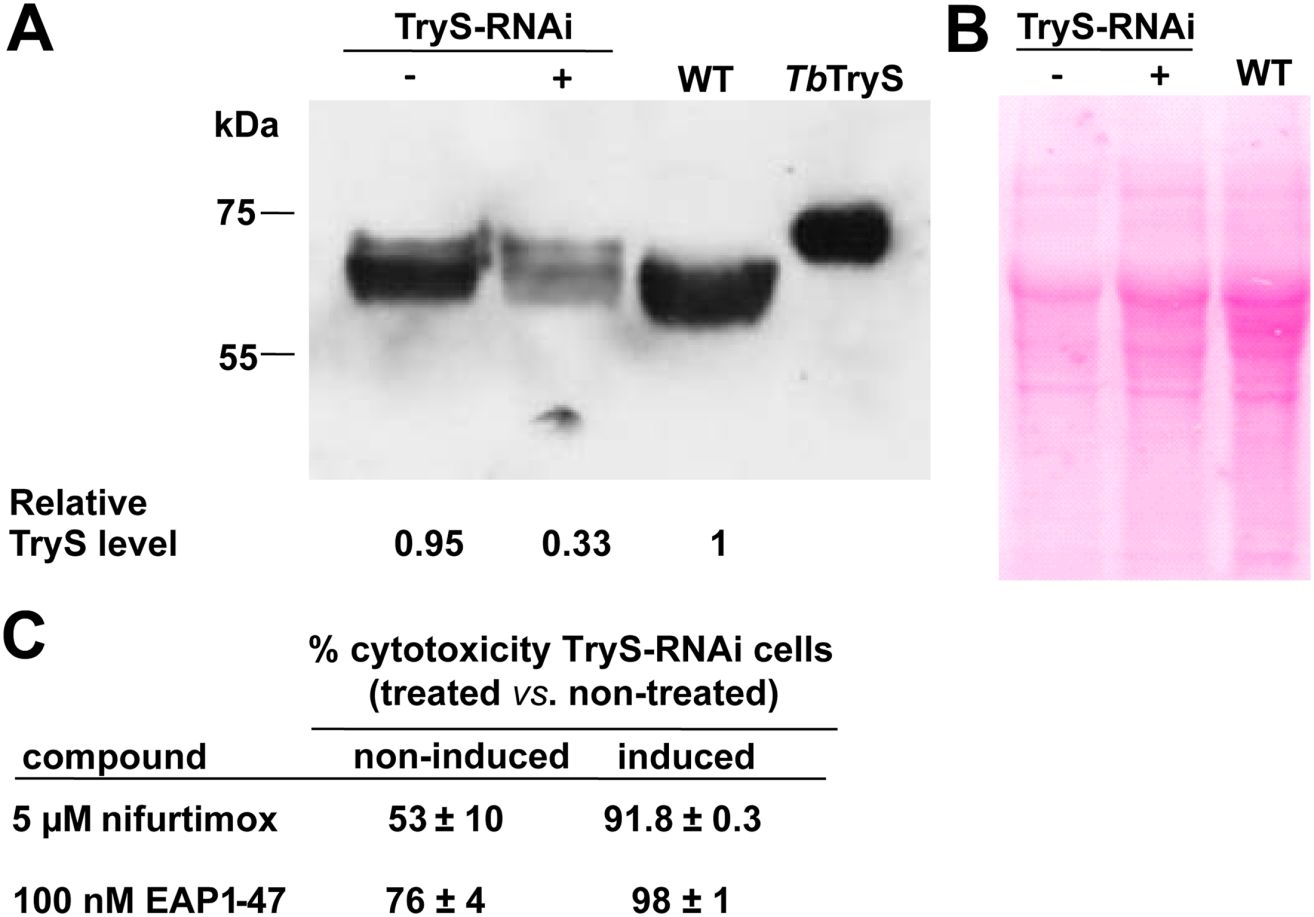
Biological activity of compounds against infective *Trypanosoma brucei brucei* with downregulated expression of trypanothione synthetase (TryS). **A)** Western blot analysis of cell extracts from 2x10^7^ *T*. *b*. *brucei* from the wildtype (WT), 48 h tetracycline-induced (+) and non-induced (-) TryS-RNAi cell line. Two hundred ng of recombinant *Tb*TryS was loaded as control. Bands from the molecular weight marker are indicated on left. The picture at the bottom shows the abundance of TryS for each condition as estimated by densitometry and expressed relative to the level of the WT cell line. **B)** Ponceau staining of the Western blot membrane that served as normalization control of protein load for each condition. **C)** Cytotoxicity (%) ± S.D. (n = 2) for tetracycline-induced (+) and non-induced (-) TryS-RNAi *T*. *b*. *brucei* treated with 5 μM nifurtimox or 100 nM EAP1-47.

In order to get an insight into the on-target activity of promising compounds, the intracellular thiol content of WT parasites exposed for 24 h to EAP1-47 and MOL2008 was determined. For bloodstream *T*. *b*. *brucei* treated with 50 nM EAP1-47, T(SH)_2_ content decreases by 28% while GSH level increases by 39% with respect to untreated cells, which overall resembles the metabolite changes observed for parasites with 48 h RNAi-downregulated expression of TryS (S11 Table and [16]). A compound acting on T(SH)_2_ metabolism is expected to display an increased cytotoxicity against parasites depleted in TryS. The cytotoxic effect of 100 nM EAP1-47 towards trypanosomes with a TryS content that is 1/3 of that corresponding to non-induced RNAi or wild-type cells was slightly increased (1.3-fold; Fig 5). For comparison, nifurtimox, a drug inducing thiol depletion [73], added at 5 μM (EC_50_ determined for WT cells) displayed a 1.8-fold increased potency towards TryS-depleted parasites. Strikingly, identical assays performed with the chemically-related and trypanosome-selective compounds EAP1-63 and APC1-99 did not show differences in the potency of these compounds towards WT or TryS-depleted parasites. Taking together, these results shows that EAP1-47 is interfering with T(SH)_2_ biosynthesis, although the almost two order of magnitude difference between *Tb*TryS inhibition and EC_50_ indicates that the compound has also other molecular targets *in vivo*. In contrast, the lack of enhanced cytotoxicity displayed by EAP1-63 and APC1-99 towards the TryS-RNAi induced cell line, is an strong indication that their potent trypanocidal activity is unrelated to interference with T(SH)_2_ metabolism.

On the other hand, *L*. *infantum* promastigotes in the log growth phase exposed to MOL2008 at its EC_50_ (12 μM) for 24 h presented a marked decrease in the intracellular pool of thiols. From two independent experiments, T(SH)_2_ and GSH content decreases ≥ 90% and > 30%, respectively, upon MOL2008 treatment (S11 Table). Interestingly and at variance with the metabolic changes observed for the genetic (RNAi) or chemical (EAP1-47) silencing of TryS in *T*. *b*. *brucei*, inhibition of TryS by MOL2008 did not lead to accumulation of the substrate GSH in *L*. *infantum*. Further experiments are required to establish whether the reduction on GSH level is caused by paullone-mediated inhibition of any of the two ATP-dependent enzymes in charge of glutathione synthesis or is a consequence of a species-specific regulatory mechanism triggered by T(SH)_2_ depletion.

## Discussion

The screening of a compound library consisting of 7 major chemical scaffolds and several singletons against TryS from three major pathogenic species of trypanosomatids at near physiological concentration of substrates led to the identification of 15 inhibitor molecules with μM (**APPDA**, **BDA**, **BZ**, **AI**) and sub-μM (**AI**) potency and a remarkable species-specificity for the molecular target. Despite the high sequence identity between tri-tryp TryS (e.g. the amino acid sequence identity is 71.6% for *Tb*TryS/*Tc*TryS, 64.7% for *Tb*TryS/*Li*TryS and 63.4% for *Tc*TryS/*Li*TryS) and the almost strict conservation of residues involved in substrate binding [20, 21], only 4 compounds were able to target TryS from different species, with only one of them (a **BDA**) inhibiting all three TryS with moderate potency (50% at 30 μM). This behavior highlights the existence of structural differences between tritryp TryS that determine their specificity for ligands. In support of this observation, single aminoacid substitutions in the homologue enzyme from *C*. *fasciculata* were previously reported to produce drastic changes in enzyme activity [23, 36] and our comparative analysis of the kinetic parameters of tritryp TryS obtained under similar assay conditions reveals considerable differences between species.

Early investigations have shown that substitutions at different positions of the paullone scaffold yield nM inhibitors of TryS from *C*. *fasciculata* or *L*. *infantum* (*N*^5^-substituted **AI**) [10, 20] and potent anti-*T*. *b*. *brucei* agents with moderate inhibition towards *Tb*TryS (11-substituted 4-azapaullone) [56]. By extending the analysis of **AI** activity towards tritryp TryS, we have confirmed that the inclusion of a *N*-[2-(methylamino)ethyl] acetamide in position *N*^*5*^ confers SP-competitive inhibition of *Li*TryS activity, since compounds lacking this substitution were almost inactive. From the tritryp TryS evaluated here, *Li*TryS presented the higher K_M_ for SP, which indicates the enzyme binds less efficiently this substrate probably due to an unfavorable conformation of the polyamine binding site. However, the shape adopted by the SP-binding site of *Li*TryS appears suitable for accommodating the *N*^5^-substituent present in MOL2008 and FS554. In support of this hypothesis, the trypanosomal TryS displayed a 2- to 6-fold lower K_M_ values for SP and a marked refractoriness to inhibition by *N*^5^-substituted paullones (> 200-fold higher IC_50_). The opposite behavior was observed for prochlorperazine, a SP-competitive inhibitor of *Tb*TryS [18]. In this case, the degree of enzyme inhibition was inversely proportional to the SP K_M_ value for each TryS, with *Tb*TryS being the most sensitive to inactivation followed by *Tc*TryS, whereas *Li*TryS was refractory to inactivation (300 μM prochlorperazine) even at a sub-K_M_ concentration of SP (240 μM *vs*. K_M_ of 1335 μM). Altogether, these data strongly point to the existence of remarkable differences in the polyamine binding site of tritryp TryS that should be carefully considered for the design of new **AI** with multi-species activity. In this respect, our study also demonstrated that in spite of the potent anti-*T*. *b*. *brucei* activity [56], **AI** substituted with α,β-unsaturated carbonyl chains in position 9 or 11 should be disregarded as ligands for future optimization because of their null to marginal anti-TryS activity.

Both *N*^5^-substituted paullones identified as potent *Li*TryS inhibitors displayed an IC_50_ slope (Hill coefficient) below the unity (~0.35), which suggests binding of the inhibitor to non-equivalent binding pockets or to partitioning of the compounds into an inactive, less potent or inaccessible form at higher concentrations [74]. Since both paullones displayed good solubility in the concentration range tested (~1 nM-50 μM) and produced 100% *Li*TryS inhibition, we ruled out insolubility or aggregation phenomena as responsible of this behavior. On the other hand, the dose-response plots do not show the presence of a second inflection point that would be indicative of a second binding pocket for paullones. TryS are monomeric but mechanistically complex (trisubstrate) enzymes [23, 24] whose substrate binding sites display large conformational changes during catalysis [21, 75, 76], hence, it is possible that the low Hill slopes reflect an equilibrium between two or more forms of the enzyme that interact differentially with the paullones. Further mechanistic studies are needed to shed light on this issue.

From this screening, MOL2008 was the most potent inhibitor identified for *Li*TryS that, in addition, shows potential for further optimization of its anti-trypanosomal TryS activity (IC_50_ ~30 μM). The related paullone FS554 was chemically validated in a previous study using transgenic cell lines of *L*. *infantum* [20]. Here we show that MOL2008 is almost one order of magnitude more potent towards *L*. *infantum* promastigotes than FS554 and, more importantly, that it targets *in vivo* trypanothione biosynthesis. The future design of MOL2008 analogues should also aim at improving its biological properties, which according to our data are far from optimal (EC50 in the μM range and SI ≤ 2) and suggest off-target effects in host cells.

Precedent studies demonstrated that disustituted polyamines are potent antiproliferative agents that interfere with the polyamine metabolism of Plasmodium and African trypanosomes [47–50]. On the basis that the substituted diamine moiety may eventually be recognized as ligand by TryS, a collection of simplified derivatives was screened for their anti-synthetase activity. Two **BDA** (EAP1-47 and APC1-111) displayed inhibitory activity, albeit moderate (IC_50_ ~ 30 μM), against multiple TryS. SAR analysis revealed the need to fulfill a specific steric demand on the enzyme interacting pocket. A rational optimization of these compounds seems difficult, since the screening performed here included a wide diversity of derivatives (mono- and di-substituted, with bulky groups or halogen atoms, and with linker of different length) that yielded only weak TryS inhibitors. Nevertheless, as in a fragment-based approach, certain moieties of the most active **BDA** can be selected as substituents of novel or known TryS inhibitors.

With few exceptions, most APC derivatives tested here were previously shown to display low to sub-μM potency towards infective *T*. *b*. *brucei* [46]. Our study confirmed and disclosed the potent (EC_50_ 15–280 nM) and selective (SI = 10–164) anti-parasitic activity of APC analogues and of two halogenated **BDA** from the EAP series, respectively, all of which exherted a moderate inhibition of *Tb*TryS (IC_50_ ~ 30 μM). Interestingly, EAP1-47 induced changes in the intracellular pool of low molecular thiols that overall resembled those triggered by RNAi-mediated silencing of TryS [16] and displayed a moderate increase in its cytotoxicity towards TryS-defficient cells that was not paralleled by the more potent and selective molecules APC1-99 and EAP1-63. Nonetheless, as pointed out before, the higher *in vivo* (anti-trypanosomal activity) vs. *in vitro* (*Tb*TryS inhibition) activity of EAP1-47 suggests that this compound is targeting other essential molecular target(s) in addition to TryS.

The positive loop of substrate inhibition of TryS by GSH has a direct impact on the design of inhibitors. On the one hand, it suggests that groups competing with GSH can be excluded from the ligand scaffold to avoid unnecessary substrate competition and to reduce the molecular mass of the inhibitor. On the other hand, it shows that full inhibition of TryS is not required because the phenomenon of substrate inhibition will amplify *in vivo* the inactivation of the enzyme, which represents a pharmacological advantage of this molecular target.

The four **APPDA** identified as moderate inhibitors of, preferentially, the *T*. *cruzi* TryS, are structuraly related to compounds originally designed as inhibitors of bacterial D-Ala:D-Ala ligase. Interestingly, these derivatives presented a 10-fold lower IC_50_ towards the trypanosomal enzyme than the analogue compounds against the corresponding bacterial target (best IC_50_ = 260 μM) [43]. The **APPDA** may serve as primary scaffolds to develope analogues with improved anti-TryS activity and, consequently, superior selectivity index. *Tc*TryS, and to minor extent *Li*TryS, was target of inhibition by a **BZ** substituted with an imidazolone. A **BZ** with an additional imidazolone group retained activity against *Tc*TryS but enhanced ATP hydrolysis by *Li*TryS and *Tb*TryS, indicating that these ligands present a molecular pattern recognized by the active site of the enzymes that should be further investigated.

Our work also disclosed several compounds enhancing ATP consumption by trypanosomal TryS. Adding value to the existence of distinctive structural features between tritryp TryS, *Tb*TryS was targeted specifically by large heterocyclic compounds from the **BBHPP** whereas *Tc*TryS was the most promiscuous enzyme, being activated by a **PD**, an **APPDA** and the **BZ** mentioned above. A single compound, a bis-imidazolone **BZ**, increased *Li*TryS activity. According to our results, the activation of ATPase activity in trypanosomal TryS by these compounds takes place only when both co-subtrates, namely GSH and SP, are present in the reaction. More detailed studies are required to establish whether such allosteric activation of TryS is paralleled by an increased production of T(SH)_2_. Compounds promoting the non-productive consumption of ATP are attractive candidates for biological evaluation, because the metabolic outcome of their action should be similar to that of true enzyme inhibitors.

In summary, this study: (i) led to the identification and on-target validation of a potent inhibitor of leishmanial TryS and of chemical scaffolds that can be further developed into inhibitors of wide spectrum against TryS from different trypanosomatid species, (ii) highlights the existence of remarkable kinetic and structural differences between tritryp TryS that prompt to obtain 3D structures of TryS from different trypanosomatids in order to guide a structure-based rationale design of multi-TryS inhibitors; iii) demonstrates the relevance of running a HTS under near physiological concentration of subtrates to minimize the possibility to detect weak competitive inhibitors with low potential to act *in vivo*

## Supporting Information

S1 Fig Kinetic characterization of tritryp trypanothione synthetase (TryS). The plots of velocity (v) / [E](s^-1^) *vs*. different substrate or product concentrations (μM) are shown for: **A)** *Trypanosoma cruzi* trypanothione synthetase (*Tc*TryS) with a.1) spermidine (SP), a 2) ATP, a.3) glutathione (GSH) and a.4) ADP; **B)** *Trypanosoma brucei* trypanothione synthetase (*Tb*TryS) with b.1) SP; b.2) ATP; b.3) GSH, and b.4) ADP; **C)** *Leishmania infantum* trypanothione synthetase (*Li*TryS) with c.1) SP, c.2) ATP, c.3) GSH, and c.4) ADP. See Materials and Methods, S1 Text and S8 Table for details about assay conditions.
(TIF)

S2 Fig Sensitivity and linearity of the end-point trypanothione synthetase (TryS) assay. In a total volume of 50 μL containing all assay reagents [150 μM ATP, 2 mM spermidine (SP), 250 μM glutathione (GSH), 5 mM DTT, 10 mM MgSO_4_, 0.5 mM EDTA, 100 mM HEPES pH 7.4, 9 mM NaCl and 10% v/v DMSO] except TryS, K_2_HPO_4_ was added at concentrations ranging from 1.25 to 140 μM. Two hundred μL BIOMOL GREEN reagent were added per well and the colorimetric reaction was allowed to develop for 20 min. The absorbance at 650 nm was measured in each well using a MultiScan EX plate reader (Thermo SCIENTIFIC). The mean A_650 nm corr_ values ± 2 S.D. BIOMOL GREEN signal is plotted against [K_2_HPO_4_]. A linear regression model based on the least square method was fitted for K_2_HPO_4_ concentrations from 0 to 50 μM.
(TIF)

S3 Fig Evaluation of interference with colorimetric reaction by assay’ reagents. The corrected absorbance of BIOMOL GREEN at 650 nm (A_650 nm corr_) is ploted against different concentrations of: **A)** DTT (mM) for a reaction containing 20 μM K_2_HPO_4_ in 150 μM ATP, 2 mM spermidine (SP), 250 μM glutathione (GSH), 10% v/v DMSO and 9 mM NaCl. The A_650 nm corr_ is expressed as percentage relative to A_650 nm corr_ at 5 mM DTT; **B)** SP (mM) for a reaction containing 20 μM K_2_HPO_4_, 150 μM ATP, 2 mM SP, 250 μM GSH, 5 mM DTT, 10% v/v DMSO and 9 mM NaCl. The A_650 nm corr_ is expressed as percentage relative to A_650 nm corr_ at SP 2 mM; **C)** glycerol (%) for a reaction containing 20 μM K_2_HPO_4_, 150 μM ATP, 2 mM SP, GSH 250 μM, 5 mM DTT and 9 mM NaCl. The A_650 nm corr_ is expressed as percentage relative to A_650 nm corr_ at 0% v/v glycerol; **D)** GSH (mM) for a reaction containing 20 μM K_2_HPO_4_, 150 μM ATP, DTT 5 mM, 10% v/v DMSO, 9 or 30 mM NaCl, and 0 or 4% v/v glycerol. The A_650 nm corr_ is expressed as percentage relative to A_650 nm corr_ at 0% v/v glycerol, 0 mM NaCl and 2.5 mM GSH (black squares), 4% v/v glycerol and 0 mM NaCl (red circles), 4% v/v glycerol and 30 mM NaCl (blue triangle) and 4% v/v glycerol and 9 mM NaCl (green triangle).
(TIF)

S4 Fig Time-dependent linearity of the screening assay. Pi production (A_650 nm corr_) was monitored at different time points (min) for recombinant *Leishmania infantum* trypanothione synthetase, *Li*TryS (a, b), *Trypanosoma cruzi* trypanothione synthetase, *Tc*TryS (c, d) and *Trypanosoma brucei* trypanothione synthetase, *Tb*TryS (f, g). The assays were conducted at RT in a total reaction volume of 50 μL containing 150 μM ATP, 2 mM SP, 5 mM DTT, 10 mM MgSO_4_, 0.5 mM EDTA, 100 mM HEPES pH 7.4, 10% v/v DMSO and 9 mM NaCl, with variable concentrations of gluthatione (GSH) and enzyme units according to the TryS species: 250 μM GSH and 2.3 x 10^−5^ μmol.min^-1^.mL^-1^ for *Li*TryS, 570 μM GSH and 3.5 x 10^−6^ μmol.min^-1^.mL^-1^ for *Tc*TryS, and 50 μM GSH and 1.5 x 10^−5^ μmol.min^-1^.mL^-1^ for *Tb*TryS. Blanks lacking enzyme were prepared for each condition. The TryS reaction was stopped at different time points by adding 200 μL BIOMOL GREEN reagent. The plates were incubated for 20 min at RT and A_650 nm_ measured using a MultiScan EX plate reader (Thermo SCIENTIFIC). The mean A_650 nm corr_ ± 2 S.D. of BIOMOL GREEN signal is plotted *vs*. time. A linear regression model based on the least square method was applied to each plot to estimate the linearity range.
(TIF)

S5 Fig Activity profile for *N*,*N'*-bis(3,4-substituted-benzyl) diamine derivatives (BDA). All compounds were evaluated at 30 μM against *Trypanosoma cruzi* trypanothione synthetase, *Tc*TryS (*Tc*), *Leishmania infantum* trypanothione synthetase, *Li*TryS (*Li*) and *Trypanosoma brucei* trypanothione synthetase, *Tb*TryS (*Tb*). TryS activity is expressed as percentage (see S3 Table). “n” denotes the number of carbons in the linker between both nitrogens. **A)** Oxygen substituted (OMe: methoxy, OBn: O-benzyl, OH: hydroxyl) **BDA** with the number indicating the position of the substitution in the phenyl ring. **B)** Halogenated (F, Cl and Br) and alkylated (iPr: isopropyl) **BDA**.
(TIF)

S1 Table APPDA, 6-arylpyrido[2,3-*d*]pyrimidine-2,7-diamine derivatives. (DOCX)

S2 Table BBHPP, 1-(benzo[*d*]thiazol-2-yl)-4-benzoyl-3-hydroxy-5-phenyl-1*H*-pyrrol-2(5*H*)-one derivatives. (DOCX)

S3 Table BDA, *N*,*N'*-bis(3,4-substituted-benzyl)diamine derivatives. (DOCX)

S4 Table BZ, benzofuroxan derivatives. (DOCX)

S5 Table AI, 4,5-dihydroazepino[4,5-*b*]indol-2(1*H*,3*H*,6*H*)-one derivatives, 4-azapaullones derivatives. (DOCX)

S6 Table PD, 1*H*-purine-2,6(3*H*,7*H*)-dione derivatives. (DOCX)

S7 Table AOCA, 2-aminooxazole-5-carboxamide derivatives. (DOCX)

S8 Table Kinetic characterization conditions for recombinant TryS. (DOCX)

S9 Table SP-competitive inhibition of *Li*TryS by MOL2008. (DOCX)

S10 Table Characterization of compounds enhancing tritryp trypanothione synthetase (TryS) activity. (DOCX)

S11 Table Analysis of intracellular thiols in bloodstream *T*. *b*. *brucei* and *L*. *infantum* promastigotes. (DOCX)

S1 Text Supporting Information. (DOCX)

## References

[1] Barrett MP, Croft SL. Management of trypanosomiasis and leishmaniasis. Br Med Bull. 2012; 104: 175–96. 10.1093/bmb/lds031 23137768 PMC3530408

[2] Gadelha C, Holden JM, Allison HC, Field MC. Specializations in a successful parasite: What makes the bloodstream-form African trypanosome so deadly?. Mol Biochem Parasitol. 2011; 179: 51–58. 10.1016/j.molbiopara.2011.06.006 21763356

[3] Nagajyothi F, Machado FS, Burleigh BA, Jelicks LA, Scherer PE, Mukherjee S, et al Mechanisms of *Trypanosoma cruzi* persistence in Chagas disease. Cell Microbiol. 2012; 14: 634–643. 10.1111/j.1462-5822.2012.01764.x 22309180 PMC3556388

[4] Filardi LS, Brener Z. Susceptibility and natural resistance of *Trypanosoma cruzi* strains to drugs used clinically in Chagas disease. Trans R Soc Trop Med Hyg. 1987; 81: 755–759. 3130683 10.1016/0035-9203(87)90020-4

[5] Croft SL, Sundar S, Fairlamb AH. Drug Resistance in Leishmaniasis. Clin Microbiol Rev. 2006; 19: 111–126. 16418526 10.1128/CMR.19.1.111-126.2006 PMC1360270

[6] Graf FE, Ludin P, Wenzler T, Kaiser M, Brun R, Pyana PP, et al Aquaporin 2 mutations in *Trypanosoma brucei gambiense* field isolates correlate with decreased susceptibility to pentamidine and melarsoprol. PLoS Negl Trop Dis. 2013; 7(10): e2475 10.1371/journal.pntd.0002475 24130910 PMC3794916

[7] Stewart ML, Burchmore RJS, Clucas C, Hertz-Fowler C, Brooks K. et al Multiple genetic mechanisms lead to loss of functional TbAT1 expression in drug-resistant trypanosomes. Eukaryot Cell. 2010; 9: 336–343. 10.1128/EC.00200-09 19966032 PMC2823006

[8] Patterson S, Wyllie S. Nitro drugs for the treatment of trypanosomatid diseases: past, present, and future prospects. Trends Parasitol. 2014; 30: 289–298. 10.1016/j.pt.2014.04.003 24776300 PMC4045206

[9] Balasegaram M, Harris S, Checchi F, Ghorashian S, Hamel C, Karunakara U. Melarsoprol versus eflornithine for treating late-stage Gambian trypanosomiasis in the Republic of the Congo. Bull World Health Organ. 2006; 84: 783–791. 17128358 10.2471/blt.06.031955 PMC2627491

[10] Jäger T (Editor), Koch O (Editor), Flohé L. (Editor), Selzer PM (Series Editor). Trypanosomatid Diseases: Molecular Routes to Drug Discovery (Drug Discovery in Infectious Diseases). Oxford, UK: Wiley-Blackwell; 2013.

[11] Berriman M, Ghedin E, Hertz-Fowler C, Blandin G, Renauld H, Bartholomeu DC, et al The genome of the African trypanosome *Trypanosoma brucei*. Science. 2005; 309: 416–422. 16020726 10.1126/science.1112642

[12] El-Sayed NM, Myler PJ, Bartholomeu DC, Nilsson D, Aggarwal G, Tran AN, et al The genome sequence of *Trypanosoma cruzi*, etiologic agent of Chagas disease. Science. 2005; 309: 409–415. 16020725 10.1126/science.1112631

[13] Ivens AC, Peacock CS, Worthey EA, Murphy L, Aggarwal G, Berriman M, et al The genome of the kinetoplastid parasite, *Leishmania major*. Science. 2005; 309: 436–442. 16020728 10.1126/science.1112680 PMC1470643

[14] Krauth-Siegel RL, Comini M. Redox control in trypanosomatids, parasitic protozoa with trypanothione-based thiol metabolism. Biochim Biophys Acta. 2008; 1780: 1236–1248. 10.1016/j.bbagen.2008.03.006 18395526

[15] Manta B, Comini M, Medeiros A, Hugo M, Trujillo M, Radi R. Trypanothione: A unique bis-glutathionyl derivative in trypanosomatids. Biochim Biophys Acta. 2013; 1830: 3199–3216. 10.1016/j.bbagen.2013.01.013 23396001

[16] Comini M, Guerrero SA, Haile S, Menge U, Lünsdorf H, Flohé L. Validation of *Trypanosoma brucei* trypanothione synthetase as drug target. Free Radic Biol Med. 2004; 36: 1289–1302. 15110394 10.1016/j.freeradbiomed.2004.02.008

[17] Wyllie S, Oza SL, Patterson S, Spinks D, Thompson S, Fairlamb AH. Dissecting the essentiality of the bifunctional trypanothione synthetase-amidase in *Trypanosoma brucei* using chemical and genetic methods. Mol Microbiol. 2009; 74: 529–540. 10.1111/j.1365-2958.2009.06761.x 19558432 PMC2784880

[18] Torrie LS, Wyllie S, Spinks D, Oza SL, Thompson S, Harrison JR, et al Chemical validation of trypanothione synthetase a potential drug target for human trypanosomiasis. J Biol Chem. 2009; 284: 36137–36145. 10.1074/jbc.M109.045336 19828449 PMC2794729

[19] Spinks D, Torrie LS, Thompson S, Harrison JR, Frearson JA, Read KD, et al Design, synthesis and biological evaluation of *Trypanosoma brucei* trypanothione synthetase inhibitors. Chem Med Chem. 2012; 7: 95–106. 10.1002/cmdc.201100420 22162199 PMC3320663

[20] Sousa AF, Gomes-Alves AG, Benítez D, Comini M, Flohé L, Jaeger T, et al Genetic and chemical analysis of trypanothione biosynthesis in the glutathionylspermidine synthetase-containing parasite *Leishmania infantum* reveals that only trypanothione synthetase is essential for survival. Free Radic Biol Med. 2014; 73: 229–238. 24853758 10.1016/j.freeradbiomed.2014.05.007

[21] Fyfe PK, Oza SL, Fairlamb AH, Hunter WN. Leishmania trypanothione synthetase-amidase structure reveals a basis for regulation of conflicting synthetic and hydrolytic activities. J Biol Chem. 2008; 283: 17672–17680. 10.1074/jbc.M801850200 18420578 PMC2427367

[22] Olin-Sandoval V, González-Chávez Z, Berzunza-Cruz M, Martínez I, Jasso-Chávez R Becker I, et al Drug target validation of the trypanothione pathway enzymes through metabolic modeling. FEBS J. 2012; 279: 1811–1833. 10.1111/j.1742-4658.2012.08557.x 22394478

[23] Comini M, Menge U, Wissing J, Flohé L. Trypanothione synthesis in *Crithidia* revisited. J Biol Chem. 2005; 280: 6850–6860. 15537651 10.1074/jbc.M404486200

[24] Leroux AE, Haanstra JR, Bakker BM, Krauth-Siegel RL. Dissecting the catalytic mechanism of *Trypanosoma brucei* trypanothione synthetase by kinetic analysis and computational modelling. J Biol Chem. 2013; 288: 23751–23764. 10.1074/jbc.M113.483289 23814051 PMC3745322

[25] Oza SL, Tetaud E, Ariyanayagam MR, Warnon SS, Fairlamb AH. A single enzyme catalyses formation of trypanothione from glutathione and spermidine in *Trypanosoma cruzi*. J Biol Chem. 2002; 277: 35853–35861. 12121990 10.1074/jbc.M204403200

[26] Oza SL, Shaw MP, Wyllie S, Fairlamb AH. Trypanothione biosynthesis in *Leishmania major*. Mol Biochem Parasitol. 2005; 139: 107–116. 15610825 10.1016/j.molbiopara.2004.10.004

[27] Oza SL, Ariyanayagam MR, Aitcheson N, Fairlamb AH. Properties of trypanothione synthetase from *Trypanosoma brucei*. Mol Biochem Parasitol. 2003; 131: 25–33. 12967709 10.1016/s0166-6851(03)00176-2

[28] De Craecker S, Verbruggen C, Rajan PK, Smith K, Haemers A, Fairlamb AH. Characterization of the peptide substrate specificity of glutathionylspermidine synthetase from *Crithidia fasciculata*. Mol Biochem Parasitol. 1997; 84: 25–32. 9041518 10.1016/s0166-6851(96)02778-8

[29] Verbruggen C, De Craecker S, Rajan PK, Jiago XY, Borloo M, Smith K, et al Phosphonic acid and phosphinic acid tripeptides as inhibitors of glutathionylspermidine synthetase. Bioorg Med Chem Lett. 1996; 6: 253–258.

[30] Kwon DS, Lin CH, Chen S, Coward JK, Walsh CT, Bollinger JM Jr. Dissection of Glutathionylspermidine synthetase/ amidase from *Escherichia coli* into autonomously folding and functional synthetase and amidase domains. J Biol Chem. 1997; 272: 2429–2436. 8999955 10.1074/jbc.272.4.2429

[31] Lin C, Chen S, Kwon DS, Coward JK, Walsh CT. Aldehyde and phosphinate analogs of glutathione and glutathionylspermidine: potent, selective binding inhibitors of the *E*. *coli* bifunctional glutathionylspermidine synthetase/amidase. Chem Biol. 1997; 4: 859–866. 9384533 10.1016/s1074-5521(97)90118-6

[32] Chen S, Lin CH, Walsh CT, Coward JK. Novel inhibitors of trypanothione biosynthesis: synthesis and evaluation of a phosphinate analog of glutathionyl spermidine (GSP), a potent, slow binding inhibitor of GSP synthetase. Bioorg Med Chem Lett. 1997; 7: 505–510.

[33] Chen S, Lin CH, Kwon DS, Walsh CT, Coward JK. Design, Synthesis, and Biochemical Evaluation of phosphonate and phosphonamidate analogues of glutathionylspermidine as inhibitors of glutathionylspermidine synthetase/amidase from *Escherichia coli*. J Med Chem. 1997; 40: 3842–3850. 9371250 10.1021/jm970414b

[34] Amssoms K, Oza SL, Ravaschino E, Yamani A, Lambeir A, Rajan P, et al Glutathione-like tripeptides as inhibitors of glutathionylspermidine synthetase. Part 1: Substitution of the glycine carboxylic acid group. Bioorg Med Chem Lett. 2002: 12: 2553–2556. 12182858 10.1016/s0960-894x(02)00489-4

[35] Amssoms K, Oza S.L, Augustyns K, Yamani A, Lambeir A.M, Bal G, et al Glutathione-like tripeptides as inhibitors of glutathionylspermidine synthetase. Part 2: substitution of the glycine part. Bioorg Med Chem Lett. 2002; 12: 2703–2705. 12217358 10.1016/s0960-894x(02)00538-3

[36] Oza SL, Chen S, Wyllie S, Coward JK, Fairlamb AH. ATP-dependent ligases in trypanothione biosynthesis—kinetics of catalysis and inhibition by phosphinic acid pseudopeptides. FEBS J. 2008; 275: 5408–5421. 10.1111/j.1742-4658.2008.06670.x 18959765 PMC2702004

[37] D’Silva C, Daunes S, Rock P, Yardley V, Croft SL. Structure-activity study on the *in vitro* antiprotozoal activity of glutathione derivatives. J Med Chem. 2000; 43: 2072–2078. 10821719 10.1021/jm990259w

[38] Daunes S, D’Silva C, Kendrick H, Yardley V, Croft SL. QSAR study on the contribution of Log *P* and *E*s to the *in vitro* antiprotozoal. Activity of glutathione derivatives. J Med Chem. 2001; 44: 2976–2983. 11520206 10.1021/jm000502n

[39] Daunes S, D’Silva C. Glutathione derivatives active against *Trypanosoma brucei rhodesiense* and *T*. *brucei brucei in vitro*. Antimicrob Agents Chemother. 2002; 46: 434–437. 11796354 10.1128/AAC.46.2.434-437.2002 PMC127061

[40] Ravaschino EL, Docampo R, Rodriguez JB. Design, synthesis, and biological evaluation of phosphinopeptides against *Trypanosoma cruzi* targeting trypanothione biosynthesis. J Med Chem. 2006; 49: 426–435. 16392828 10.1021/jm050922i

[41] Stuhlmann F, Jäger T, Flohé L, Schinzer D. *N*^*5*^-substituted 2-(6-oxo-6,7-dihydro-*5H*-benzo[2,3]azepino[4,5-*b*]indol-5-yl)-acetamides for treating tropical diseases. EP 1 757 607 A1. 2007. Feb 28. http://www.google.com/patents/EP1757607A1?cl=en

[42] Haldane JBS. Enzymes. New York: Longmans, Green; 1930.

[43] Škedelj V, Arsovska E, Tomašić T, Kroflič A, Hodnik V, Hrast M, et al 6-Arylpyrido[2,3-*d*]pyrimidines as novel ATP-competitive inhibitors of bacterial D-Alanine:D-Alanine ligase. PloS One 2012; 7(8): e39922 10.1371/journal.pone.0039922 22876277 PMC3410885

[44] Miller JR, Dunham S, Mochalkin I, Banotai C, Bowman M, Buist S, et al A class of selective antibacterials derived from a protein kinase inhibitor pharmacophore. Proc Natl Acad Sci. U S A. 2009; 106: 1737–1742. 10.1073/pnas.0811275106 19164768 PMC2644107

[45] Labadie GR, Choi SR, Avery MA. Diamine derivatives with antiparasitic activities. Bioorg Med Chem Lett. 2004; 14: 615–619. 14741254 10.1016/j.bmcl.2003.11.055

[46] Caminos AP, Panozzo-Zenere EA, Wilkinson SR, Tekwani BL, Labadie GR. Synthesis and antikinetoplastid activity of a series of N,N’-substituted diamines. Bioorg Med Chem Lett. 2012; 22: 1712–1715. 10.1016/j.bmcl.2011.12.101 22248858

[47] Bitonti AJ, McCann PP, Sjoerdsma A. The effects of polyamine analogs on malaria parasites *in vitro* and *in vivo*. Adv Exp Med Biol. 1998; 250: 717–726. 10.1007/978-1-4684-5637-0_63 2475014

[48] Bitonti AJ, Dumont JA, Bush TL, Edwards ML, Stemerick DM, McCann PP, et al Bis(benzyl)polyamine analogs inhibit the growth of chloroquine-resistant human malaria parasites (*Plasmodium falciparum*) *in vitro* and in combination with a-difluoromethylornithine cure murine malaria. Proc Natl Acad Sci USA. 1989; 86: 651–655. 2463635 10.1073/pnas.86.2.651 PMC286531

[49] Bitonti AJ, Dumont JA, Bush TL, Stemerick DM, Edwards ML, McCann PP. Bis(benzyl)polyamine analogs as novel substrates for polyamine oxidase. J Biol Chem. 1990; 265: 382–388. 2294109

[50] Byers TL, Bush TL, McCann PP, Bitonti AJ. Antitrypanosomal effects of polyamine biosynthesis inhibitors correlate with increases in *Trypanosoma brucei brucei* S-adenosyl-L-methionine. Biochem J. 1991; 274: 527–533. 1672500 10.1042/bj2740527 PMC1150171

[51] Šarlauskas J, Miliukienė V, Anusevičius Ž, Misevičienė L, Krikštopaitis K, Nemeikaitė-Čėnienė A., et al Redox properties and prooxidant cytotoxicity of benzofuroxans: a comparison with nitrobenzenes, Chemija. 2009; 20: 109–115.

[52] Šarlauskas J, Anusevičius Ž, Misiūnas A. Benzofuroxan (Benzo[1,2-c]1,2,5-oxadiazole *N*-oxide) derivatives as potential energetic materials: studies on their synthesis and properties. Central European Journal of Energetic Materials. 2012; 9: 365–386.

[53] Šarlauskas J.Synthesis of energetic materials containing benzimidazole core. Proceedings of Seminar on New Trends in Research of Energetic Materials 11(part 2); 2010 p. 730–737.

[54] Šarlauskas J. Synthesis of some new heterocyclic derivatives of benzofuroxan. in Proceedings of 7^th^ Natl. Lithuanian Conference “*Chemistry*” 7; 2005. p. 104–109.

[55] Šarlauskas J, Misevičienė L, Marozienė A, Karvelis L, Stankevičiūtė J, Krikštopaitis K, et al The study of NADPH-dependent flavoenzyme-catalyzed reduction of benzo[1,2-c]1,2,5-oxadiazole *N*-oxides (Benzofuroxans). Int J Mol Sci 2014; 15: 23307–31. 10.3390/ijms151223307 25517035 PMC4284768

[56] Maiwald F, Benítez D, Charquero D, Abad Dar M, Erdmann H, Preu L, et al 9- and 11-substituted 4-azapaullones are potent and selective inhibitors of African trypanosome. Eur J Med Chem. 2014; 18: 274–283. 10.1016/j.ejmech.2014.06.020 24973661

[57] Fueller F, Jehle B, Putzker K, Lewis JD, Krauth-Siegel RL. High Throughput Screening against the peroxidase cascade of African trypanosomes identifies antiparasitic compounds that inactivate tryparedoxin. J Biol Chem. 2012; 12: 8792–8802. 10.1074/jbc.M111.338285 PMC3308743 22275351

[58] Taylor MC, Kaur H, Blessington B, Kelly JM, Wilkinson SR. Validation of spermidine synthase as a drug target in African Trypanosomes. Biochem J. 2008; 409: 563–569. 17916066 10.1042/BJ20071185 PMC2427175

[59] Coombs GH, Sanderson BE. Amine production by *Leishmania mexicana*. Ann Trop Med Parasitol. 1985; 79: 409–415. 4073994 10.1080/00034983.1985.11811939

[60] Ariyanayagam MR, Oza SL, Mehlert A, Fairlamb AH. Bis(glutathionyl)spermine and other novel trypanothione analogues in *Trypanosoma cruzi*. J Biol Chem. 2003; 278: 27612–27619. 12750367 10.1074/jbc.M302750200

[61] Zhang JH, Chung TD, Oldenburg KR. A simple statistical parameter for use in evaluation and validation of high throughput screening assays. J Biomol Screen.1999; 4: 67–73. 10838414 10.1177/108705719900400206

[62] Fairlamb AH, Henderson GB, Bacchi CJ, Cerami A. *In vivo* effects of difluoromethylornithine on trypanothione and polyamine levels in bloodstream forms of *Trypanosoma brucei*. Mol Biochem Parasitol. 1987; 24: 185–191. 3114634 10.1016/0166-6851(87)90105-8

[63] Comini MA, Dirdjaja N, Kaschel M, Krauth-Siegel RL. Preparative enzymatic synthesis of trypanothione and trypanothione analogues. Int J Parasitol. 2009; 39: 1059–1062. 10.1016/j.ijpara.2009.05.002 19477177

[64] Koenig K, Menge U, Kiess M, Wray V, Flohé L. Convenient isolation and kinetic mechanism of glutathionylspermidine synthetase from *Crithidia fasciculata*. J Biol Chem. 1997; 272: 11908–11915. 9115252 10.1074/jbc.272.18.11908

[65] Haanstra JR, van Tuijl A, van Dam J, van Winden W, Tielens AG, van Hellemond JJ, et al Proliferating bloodstream-form *Trypanosoma brucei* use a negligible part of consumed glucose for anabolic processes. Int J Parasitol. 2012; 42: 667–673. 10.1016/j.ijpara.2012.04.009 22580731

[66] Visser N, Opperdoes FR. (1980) Glycolysis in *Trypanosoma brucei*. Eur J Biochem. 1980; 103: 623–632. 6766864 10.1111/j.1432-1033.1980.tb05988.x

[67] Martins RM, Covarrubias C, Rojas RG, Silber AM, Yoshida N. Use of L-Proline and ATP production by *Trypanosoma cruzi* metacyclic forms as requirements for host cell invasion. Infect Immun. 2009; 77: 3023–3032. 10.1128/IAI.00138-09 19433547 PMC2708565

[68] Zilberstein D, Dwyer DM. Proton motive force-driven active transport of D-glucose and L-proline in the protozoan parasite *Leishmania donovani*. Proc Natl Acad Sci USA. 1985; 82:1716–1720. 2984665 10.1073/pnas.82.6.1716 PMC397343

[69] Bakker BM, Michels PAM, Opperdoes FR, Westerhoff HV. Glycolysis in bloodstream form *Trypanosoma brucei* can be understood in terms of the kinetics of the glycolytic enzymes. J Biol Chem. 1997; 272: 3207–3215. 9013556 10.1074/jbc.272.6.3207

[70] Graven P, Tambalo M, Scapozza L, Perozzo R. Purine metabolite and energy charge analysis of *Trypanosoma brucei* cells in different growth phases using an optimized ion-pair RP-HPLC/UV for the quantification of adenine and guanine pools. Exp Parasitol. 2014; 141: 28–38. 10.1016/j.exppara.2014.03.006 24657574

[71] Stoppani AO, Docampo R, de Boiso JF, Frasch AC. Effect of inhibitors of electron transport and oxidative phosphorylation on *Trypanosoma cruzi* respiration and growth. Mol Biochem Parasitol. 1980; 2: 3–21. 7007881 10.1016/0166-6851(80)90044-4

[72] Hart DT, Vickerman K, Coombs GH. A quick, simple method for purifying *Leishmania mexicana* amastigotes in large mumbers. Parasitol. 1981; 82: 345–355. 10.1017/s0031182000066889 7243344

[73] Boiani M, Piacenza L, Hernández P, Boiani L, Cerecetto H, González M, Denicola A. Mode of action of nifurtimox and N-oxide-containing heterocycles against *Trypanosoma cruzi*: is oxidative stress involved? Biochem Pharmacol. 2010; 79: 1736–1745. 10.1016/j.bcp.2010.02.009 20178775

[74] Coopeland R A (Editor). Evaluation of enzyme inhibitors in drug discovery: a guide for medicinal chemists and pharmacologists. New Jersey, USA: John Wiley & Sons, Inc.; 2005. 16350889

[75] Koch O, Cappel D, Nocker M, Jager T, Flohé L, Sotriffer CA, et al Molecular Dynamics Reveal Binding Mode of Glutathionylspermidine by Trypanothione Synthetase. PloS One. 2013; 8(2): e56788 10.1371/journal.pone.0056788 23451087 PMC3581523

[76] Pai CH, Chiang BY, Ko TP, Chou CC, Chong CM, Yen FJ, et al Dual binding sites for translocation catalysis by *Escherichia coli* glutathionylspermidine synthetase. EMBO J. 2006; 25: 5970–5982. 17124497 10.1038/sj.emboj.7601440 PMC1698887

