# Supplementary figures and images for: Identification of Novel Chemical Scaffolds Inhibiting Trypanothione Synthetase from Pathogenic Trypanosomatids
Source: PLoS Negl Trop Dis. 2016 Apr 12;10(4):e0004617. doi: 10.1371/journal.pntd.0004617 (PMC4829233; doi:10.1371/journal.pntd.0004617)

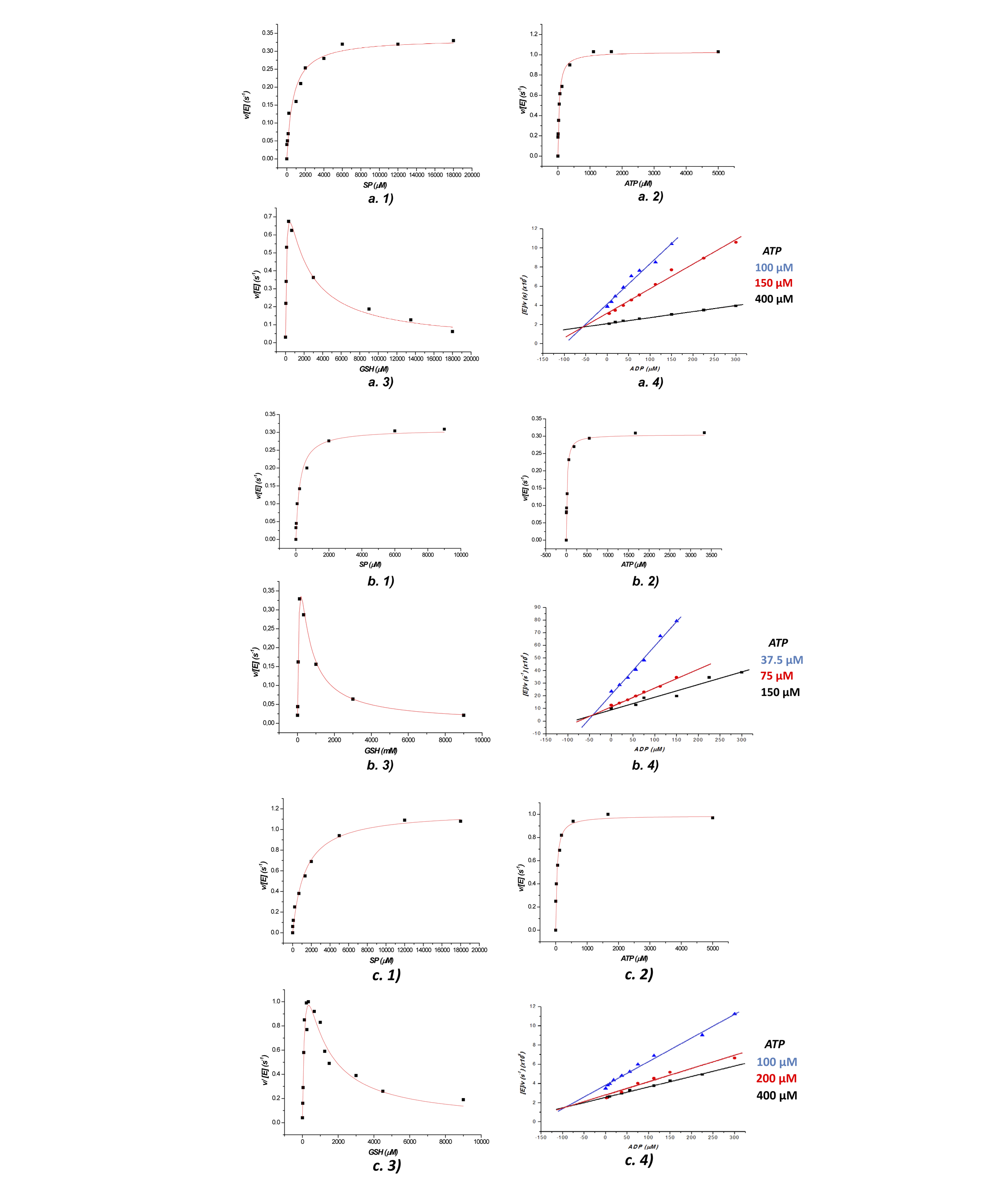

Supplement: S1 Fig — The plots of velocity (v) / [E](s-1) vs. different substrate or product concentrations (μM) are shown for: A) Trypanosoma cruzi trypanothione synthetase (TcTryS) with a.1) spermidine (SP), a 2) ATP, a.3) glutathione (GSH) and a.4) ADP; B) Trypanosoma brucei trypanothione synthetase (TbTryS) with b.1) SP; b.2) ATP; b.3) GSH, and b.4) ADP; C) Leishmania infantum trypanothione synthetase (LiTryS) with c.1) SP, c.2) ATP, c.3) GSH, and c.4) ADP. See Materials and Methods, S1 Text and S8 Table for details about assay conditions. (TIF) [file pntd.0004617.s001.tif]

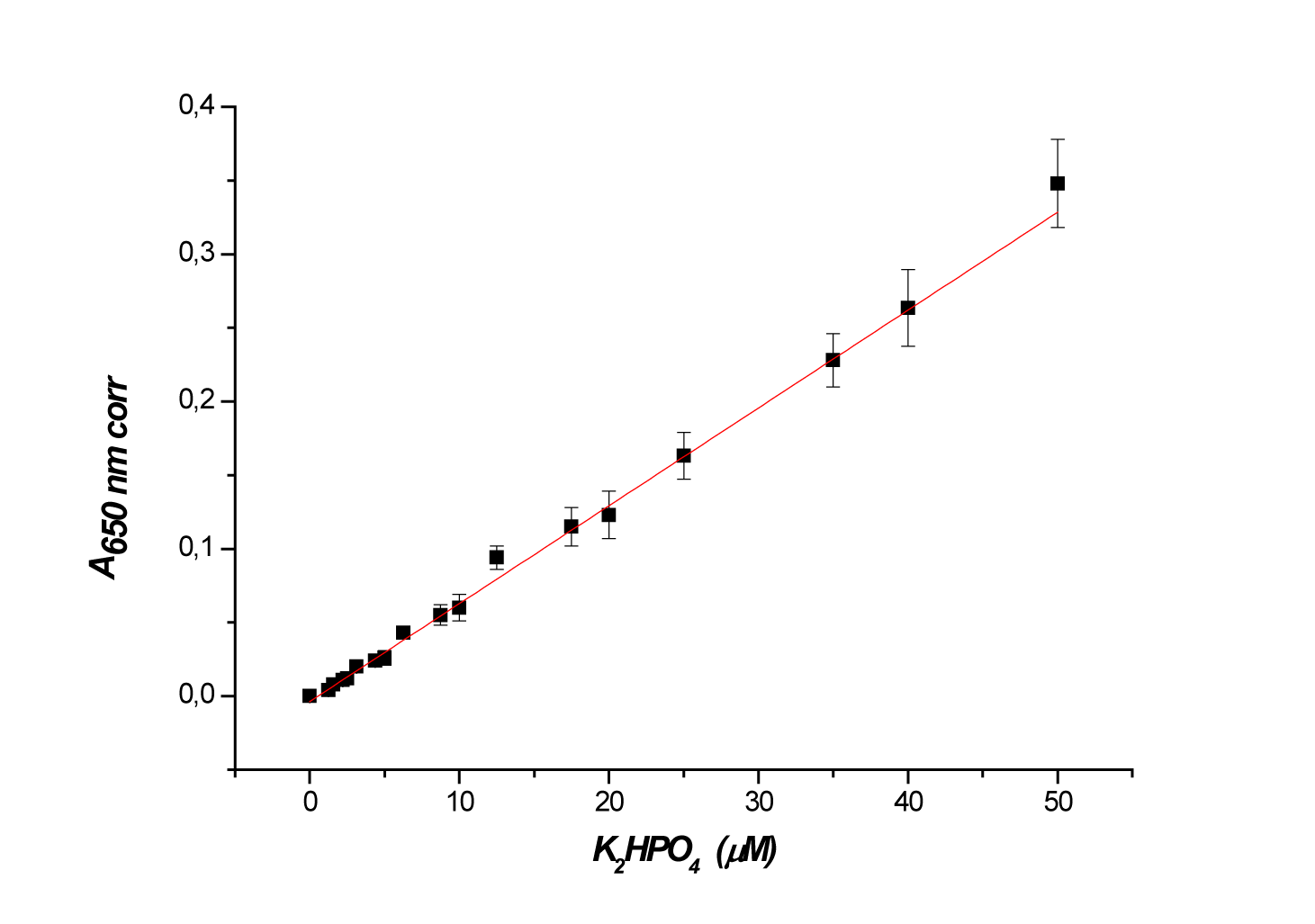

Supplement: S2 Fig — In a total volume of 50 μL containing all assay reagents [150 μM ATP, 2 mM spermidine (SP), 250 μM glutathione (GSH), 5 mM DTT, 10 mM MgSO4, 0.5 mM EDTA, 100 mM HEPES pH 7.4, 9 mM NaCl and 10% v/v DMSO] except TryS, K2HPO4 was added at concentrations ranging from 1.25 to 140 μM. Two hundred μL BIOMOL GREEN reagent were added per well and the colorimetric reaction was allowed to develop for 20 min. The absorbance at 650 nm was measured in each well using a MultiScan EX plate reader (Thermo SCIENTIFIC). The mean A650 nm corr values ± 2 S.D. BIOMOL GREEN signal is plotted against [K2HPO4]. A linear regression model based on the least square method was fitted for K2HPO4 concentrations from 0 to 50 μM. (TIF) [file pntd.0004617.s002.tif]

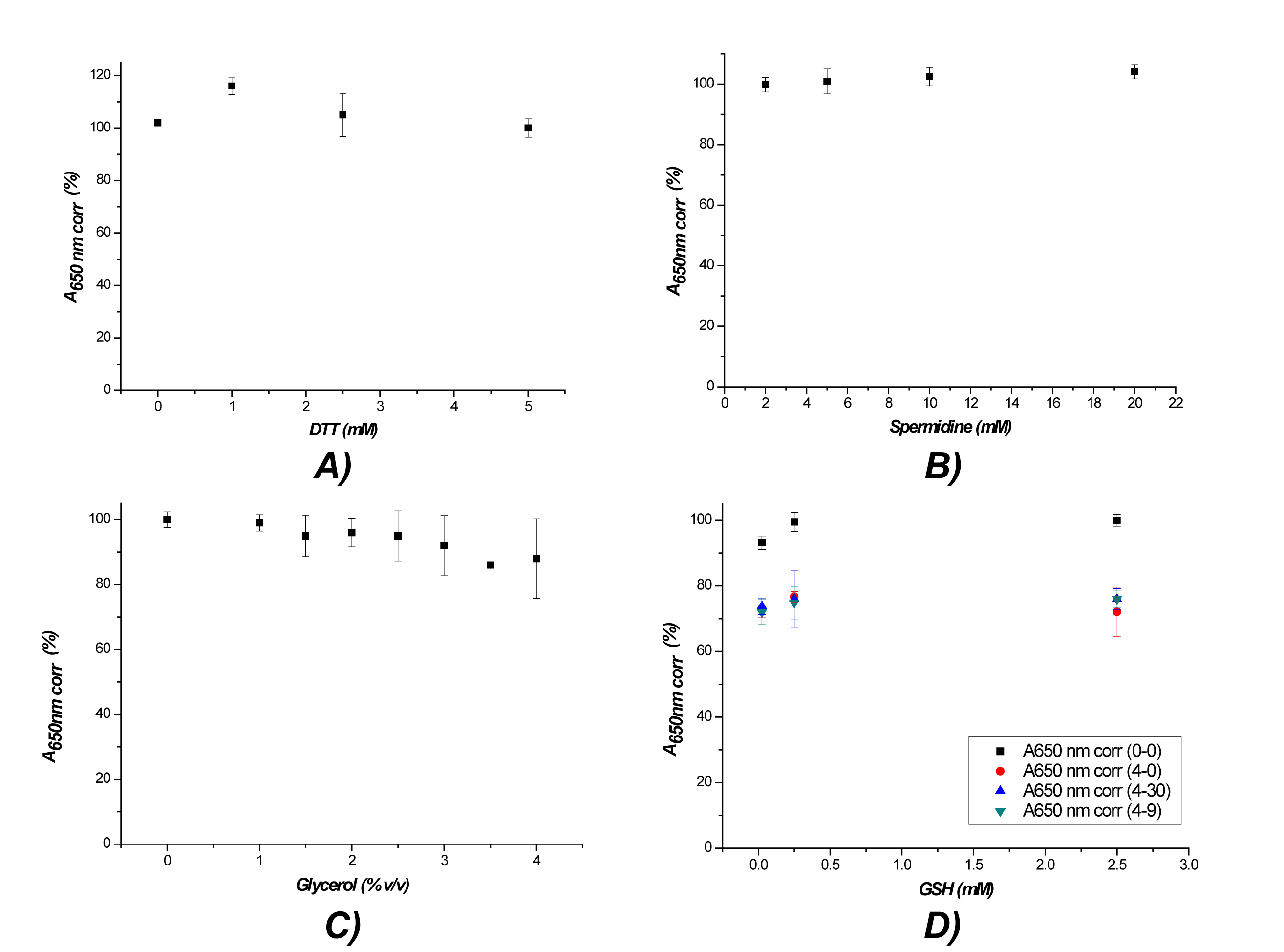

Supplement: S3 Fig — The corrected absorbance of BIOMOL GREEN at 650 nm (A650 nm corr) is ploted against different concentrations of: A) DTT (mM) for a reaction containing 20 μM K2HPO4 in 150 μM ATP, 2 mM spermidine (SP), 250 μM glutathione (GSH), 10% v/v DMSO and 9 mM NaCl. The A650 nm corr is expressed as percentage relative to A650 nm corr at 5 mM DTT; B) SP (mM) for a reaction containing 20 μM K2HPO4, 150 μM ATP, 2 mM SP, 250 μM GSH, 5 mM DTT, 10% v/v DMSO and 9 mM NaCl. The A650 nm corr is expressed as percentage relative to A650 nm corr at SP 2 mM; C) glycerol (%) for a reaction containing 20 μM K2HPO4, 150 μM ATP, 2 mM SP, GSH 250 μM, 5 mM DTT and 9 mM NaCl. The A650 nm corr is expressed as percentage relative to A650 nm corr at 0% v/v glycerol; D) GSH (mM) for a reaction containing 20 μM K2HPO4, 150 μM ATP, DTT 5 mM, 10% v/v DMSO, 9 or 30 mM NaCl, and 0 or 4% v/v glycerol. The A650 nm corr is expressed as percentage relative to A650 nm corr at 0% v/v glycerol, 0 mM NaCl and 2.5 mM GSH (black squares), 4% v/v glycerol and 0 mM NaCl (red circles), 4% v/v glycerol and 30 mM NaCl (blue triangle) and 4% v/v glycerol and 9 mM NaCl (green triangle). (TIF) [file pntd.0004617.s003.tif]

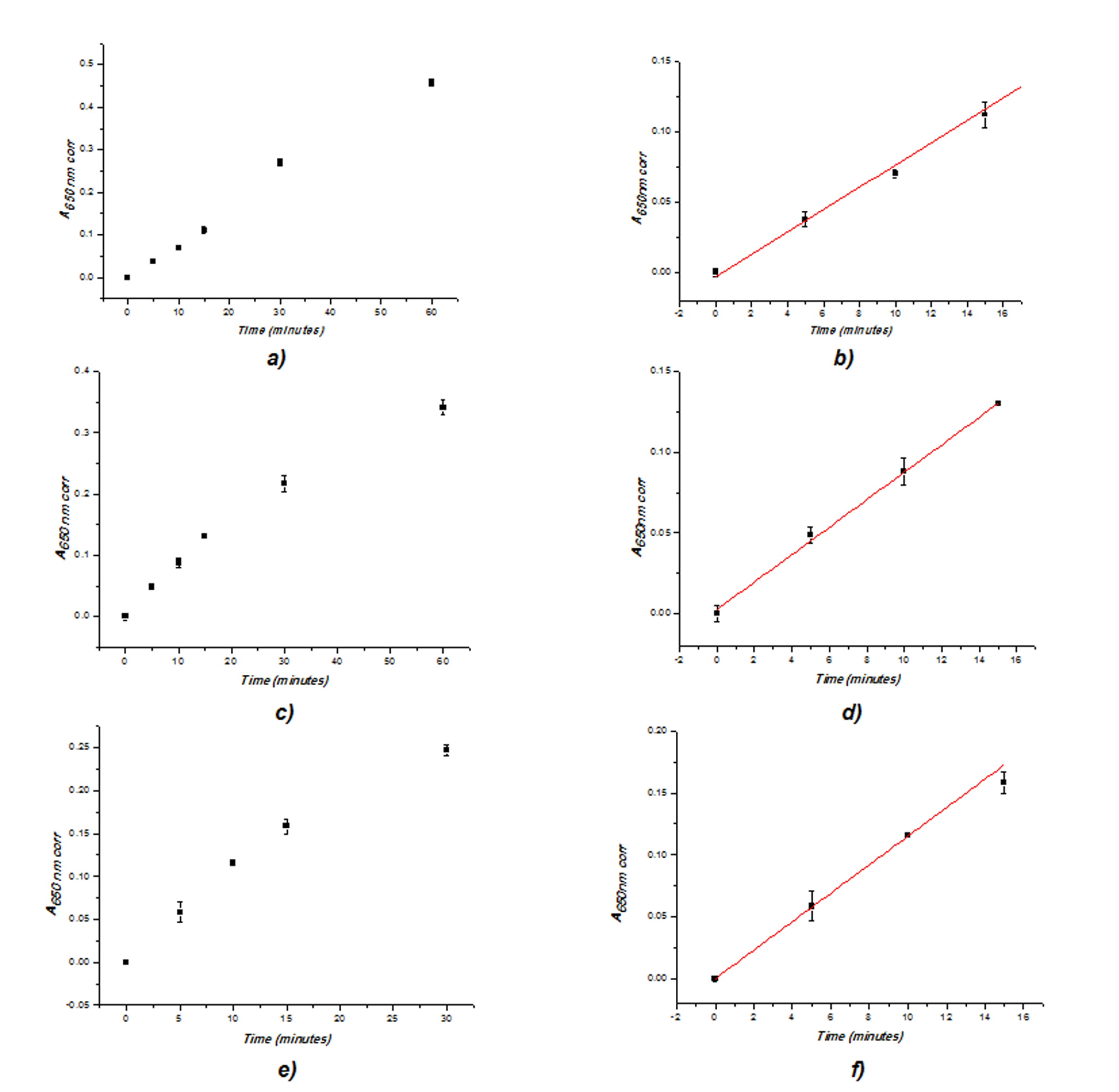

Supplement: S4 Fig — Pi production (A650 nm corr) was monitored at different time points (min) for recombinant Leishmania infantum trypanothione synthetase, LiTryS (a, b), Trypanosoma cruzi trypanothione synthetase, TcTryS (c, d) and Trypanosoma brucei trypanothione synthetase, TbTryS (f, g). The assays were conducted at RT in a total reaction volume of 50 μL containing 150 μM ATP, 2 mM SP, 5 mM DTT, 10 mM MgSO4, 0.5 mM EDTA, 100 mM HEPES pH 7.4, 10% v/v DMSO and 9 mM NaCl, with variable concentrations of gluthatione (GSH) and enzyme units according to the TryS species: 250 μM GSH and 2.3 x 10^−5^ μmol.min-1.mL-1 for LiTryS, 570 μM GSH and 3.5 x 10^−6^ μmol.min-1.mL-1 for TcTryS, and 50 μM GSH and 1.5 x 10^−5^ μmol.min-1.mL-1 for TbTryS. Blanks lacking enzyme were prepared for each condition. The TryS reaction was stopped at different time points by adding 200 μL BIOMOL GREEN reagent. The plates were incubated for 20 min at RT and A650 nm measured using a MultiScan EX plate reader (Thermo SCIENTIFIC). The mean A650 nm corr ± 2 S.D. of BIOMOL GREEN signal is plotted vs. time. A linear regression model based on the least square method was applied to each plot to estimate the linearity range. (TIF) [file pntd.0004617.s004.tif]

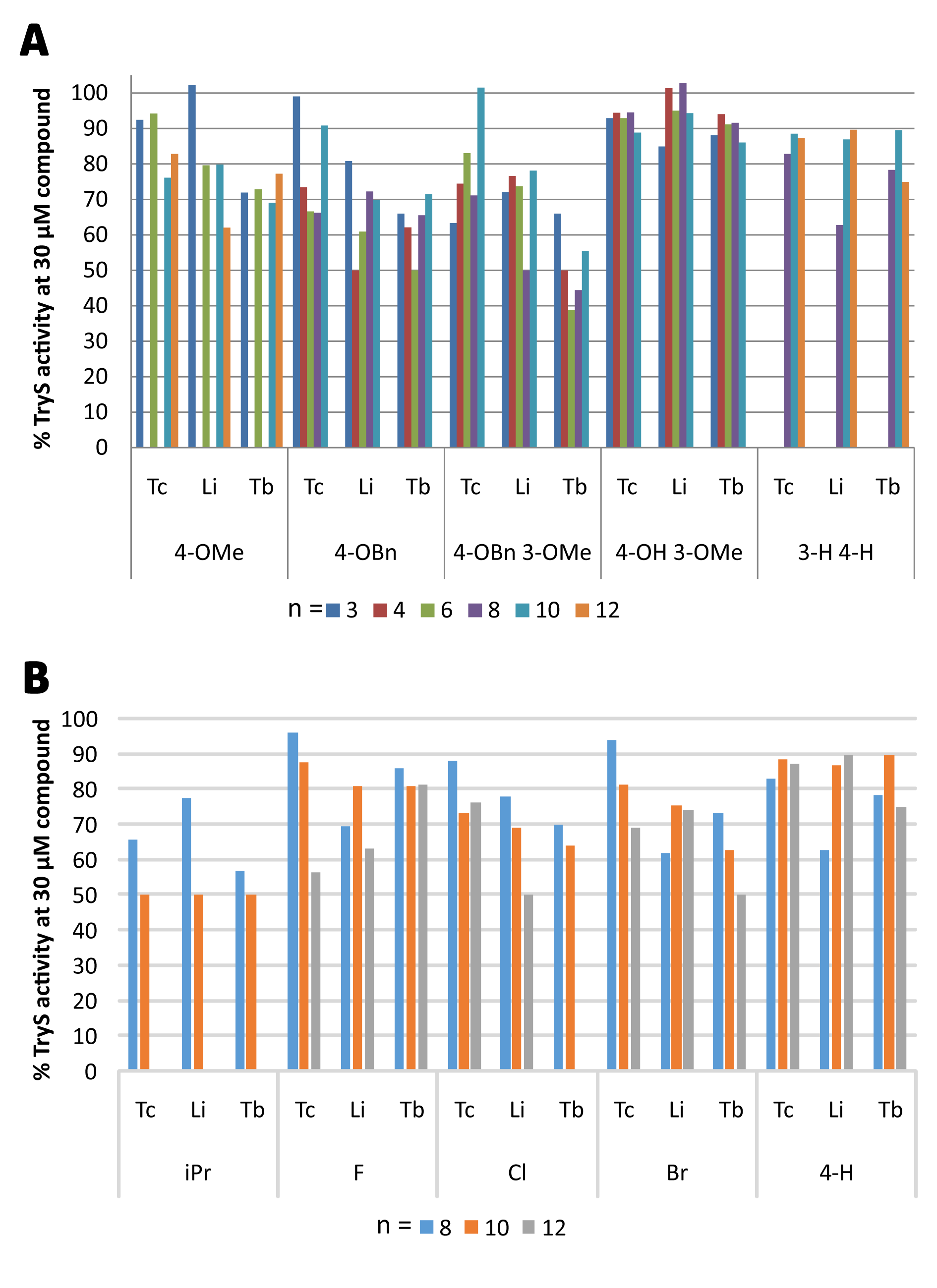

Supplement: S5 Fig — All compounds were evaluated at 30 μM against Trypanosoma cruzi trypanothione synthetase, TcTryS (Tc), Leishmania infantum trypanothione synthetase, LiTryS (Li) and Trypanosoma brucei trypanothione synthetase, TbTryS (Tb). TryS activity is expressed as percentage (see S3 Table). “n” denotes the number of carbons in the linker between both nitrogens. A) Oxygen substituted (OMe: methoxy, OBn: O-benzyl, OH: hydroxyl) BDA with the number indicating the position of the substitution in the phenyl ring. B) Halogenated (F, Cl and Br) and alkylated (iPr: isopropyl) BDA. (TIF) [file pntd.0004617.s005.tif]
