# Supplementary material for: Identification of Novel Chemical Scaffolds Inhibiting Trypanothione Synthetase from Pathogenic Trypanosomatids
Source: PLoS Negl Trop Dis. 2016 Apr 12;10(4):e0004617. doi: 10.1371/journal.pntd.0004617 (PMC4829233; doi:10.1371/journal.pntd.0004617)
Supplement: S1 Table — (DOCX) [file pntd.0004617.s006.docx]

**Table S1. APPDA, 6-arylpyrido[2,3-*d*]pyrimidine-2,7-diamine derivatives.**

|  | | | | | | |
| --- | --- | --- | --- | --- | --- | --- |
|  | **Substitutions** | | | **Activity ± 2σ^n-1^ (%); n**  **(interference factor)** | | |
| **Name** | **R_1_** | **R_2_** | **R_3_** | ***Tc*TryS** | ***Li*TryS** | ***Tb*TryS** |
| ZEA5 | H | H |  | 60.8 ± 3.9; 4 | 82.9 ± 6.4; 4 | 99.1 ± 5.0; 3 |
| *ZEA10* | H |  |  | 67.7 ± 4.0; 3 | 67.9 ± 6.4; 3 | ~ 30  (1.41) |
| *ZVR105* | H | H |  | 100.6 ± 1.6; 2 | 98.2 ± 8.2; 4 | 90.5 ± 4.5; 4 |
| *ZVR134* | H |  |  | 82.7 ± 3.4; 4 | 99.5 ± 4.0; 4 | 89.1 ± 1.3; 2 |
| *ZVR100* | H | H |  | 82.7 ± 5.3; 4 | 100.4 ± 6.8; 3 | 77.3 ± 4.8; 4 |
| *ZVR106* | H |  |  | 72.7 ± 3.1; 3 | 88.6 ± 7.4; 4 | 75.1 ± 5.4; 4 |
| *ZVR151* |  | H |  | 88.3 ± 7.2; 4 | 87.6 ± 1.9; 4 | 92.0 ± 8.5; 3 |
| *ZVR152* |  | H |  | 85.5 ± 3.4; 4 | 92.0 ± 7.4; 4 | 93.8 ± 4.0; 2 |
| *ZVR135* | H |  |  | 79.1 ± 6.3; 3 | 96.4 ± 3.9; 3 | 88.4 ± 2.0; 3 |
| *ZVR158* |  |  |  | 75.1 ± 0.8; 4 | 94.9 ± 4.9; 4 | 63.5 ± 5.7; 3 |
| *ZVR159* |  |  |  | 168.6 ± 4.1; 4 (1.10) | 100.6 ± 5.8; 4 | 81.3 ± 1.6; 3 |
| *ZVR108* | H | H |  | 85.0 ± 3.9; 4 | 103.5 ± 6.0; 4 | 92.1 ± 3.2; 4 |
| *ZEA41* | H |  |  | ~30  (0.99) | 86.4 ± 3.4; 3 | 79.1 ± 4.4; 4 |
| *ZVR110* | H |  |  | 71.7 ± 4.5; 3 | 95.5 ± 5.6; 4 | 93.8 ± 5.8; 4 |
| *ZEA40* |  |  |  | ~30  (1.02) | 84.7 ± 8.3; 4 | 74.7 ± 5.2; 4 |
| *ZVR107* | H | H |  | 84.8 ± 6.7; 3 | 104.1 ± 8.3; 4 | 88.9 ± 5.0; 4 |
| *ZVR111* | H |  |  | 80.3 ± 3.2; 4 | 93.0 ± 7.9; 3 | 92.3 ± 2.9; 4 |
| *ZVR160* | H | H |  | 73.9 ± 4.1; 3 | 103.0 ± 9.5; 4 | 79.2 ± 2.5; 4 |
| *ZVR163* | H |  |  | 75.5 ± 7.8; 4 | 99.1 ± 8.8; 4 | 92.0 ± 5.7; 3 |
| *ZVR157* | H | H |  | 77.3 ± 3.7; 4 | 79.3 ± 4.2; 3 | 80.6 ± 6.0; 4 |
| *ZVR175* | H |  |  | 81.0 ± 5.1; 3 | 88.1 ± 5.0; 3 | 71.5 ± 6.0; 4 |
| *ZEA3* | H | H |  | 64.3 ± 2.0; 3 | 99.0 ± 9.2; 3 | 85.5 ± 2.9; 3 |
| *ZVR130* |  | H |  | 91.8 ± 2.0; 4 | 100.9 ± 5.4; 4 | 96.5 ± 3.2; 3 |
| *ZEA8* | H |  |  | 104.3 ± 6.8; 2 | 90.4 ± 3.2; 4 | 77.3 ± 3.1; 2 |
| *ZVR131* |  |  |  | 97.4 ± 8.3; 4 | 108.7 ± 8.8; 3 | 89.2 ± 5.0; 3 |
| *ZVR132* |  | H |  | 87.0 ± 4.2; 4 | 99.7 ± 2.3; 4 | 75.8 ± 4.6; 3 |
| *ZEA34* | H |  |  | 80.3 ± 4.3; 3 | 99.8 ± 6.7; 3 | 83.4 ± 4.6; 3 |
| *ZVR133* |  |  |  | 90.3 ± 2.4; 4 | 94.1 ± 6.7; 3 | 69.8 ± 4.2; 4 |
| *ZEA35* |  |  |  | ~30  (1.01) | 86.2 ± 9.7; 4 | 75.7 ± 6.1; 3 |
| *ZVR140* | H | H |  | 76.8 ± 2.8; 4 | 95.4 ± 5.2; 3 | 78.4 ± 5.6; 4 |
| *ZVR141* | H |  |  | 80.5 ± 3.6; 4 | 96.4 ± 6.6; 4 | 89.3 ± 7.8; 3 |
| *ZVR150* | H | H |  | 86.7 ± 6.4; 4 | 83.3 ± 0.6; 3 | 84.7 ± 5.4; 4 |
| *ZVR156* | H |  |  | 86.4 ± 7.2; 4 | 102.9 ± 3.1; 4 | 84.2 ± 6.4; 3 |
| *ZVR165* | H | H |  | 84.8 ± 6.4; 4 | 98.4 ± 4.9; 4 | 66.6 ± 4.0; 5 |
| *ZVR167* | H |  |  | 75.3 ± 6.0; 4 | 95.2 ± 8.8; 3 | 80.0 ± 5.0; 5 |

Enzyme activity is expressed as % TryS activity ± 2σ^n-1^ and for compounds that at 30 µM inhibit TryS by 45-55%, an estimated IC_50_ value of ~30 µM is provided. For compounds affecting BIOMOL GREEN signal, the interference factor used to correct TryS activity is provided in brackets (see Materials & Methods and S1 Text). The number of assay replicates is shown after the semicolon.
