# Supplementary material for: Identification of Novel Chemical Scaffolds Inhibiting Trypanothione Synthetase from Pathogenic Trypanosomatids
Source: PLoS Negl Trop Dis. 2016 Apr 12;10(4):e0004617. doi: 10.1371/journal.pntd.0004617 (PMC4829233; doi:10.1371/journal.pntd.0004617)
Supplement: S2 Table — (DOCX) [file pntd.0004617.s007.docx]

**Table S2. BBHPP, (1-(benzo[*d*]thiazol-2-yl)-4-benzoyl-3-hydroxy-5-phenyl-1*H*-pyrrol-2(5*H*)-one derivatives).**

|  | | | | | | | | |
| --- | --- | --- | --- | --- | --- | --- | --- | --- |
|  | **Substitutions** | | | | | **Activity ±**  **2σ ^n-1^ (%); n**  **(interference factor)** | | |
| **Name** | **R_1_** | **R_2_** | **R_3_** | **R_4_** | **R_5_** | ***Tc*TryS** | ***Li*TryS** | ***Tb*TryS** |
| *AD81* |  | | OCH_3_ | H | CH_3_ | 109.7 ± 8.9; 4 | 83.9 ± 4.2; 4 | 128.2 ± 3.6 ; 8 (0.93) |
| *AD82* |  | | OH | H | CH_3_ | 103.4 ± 6.4; 2 | 86.2 ± 5.8; 4 | 107.7 ± 6.4; 4 |
| *AD83* |  | | OH | H | Cl | 93.6 ± 4.7; 3 (0.93) | 83.9 ± 3.8; 4 | 106.9 ± 4.7; 3 |
| *AD84* |  | | OCH_3_ | H | Cl | 98.7 ± 5.1; 4 | 82.1 ± 4.0; 4 | 119.0 ± 6.3; 8 (0.95) |
| *ADMRC150* |  | | F | H | Cl | 81.2 ± 4.1; 4 | 63.0 ± 6.8; 7 | 101.4 ± 8.2; 12 |
| *ADMRC151* |  | | H | H | CH_3_ | 97.0 ± 5.2; 3 | 83.1 ± 2.3; 3 | 107.7 ± 6.1; 3 |
| *ADMRC152* |  | | OH | OCH_3_ | CH_3_ | 103.1 ± 6.2; 3 | 92.5 ± 6.6; 4 | 115.4 ± 4.3; 3 |
| *ADMRC153* |  | | OCH_3_ | OCH_3_ | CH_3_ | 109.3 ± 4.7; 4 | 87.6 ± 6.0; 4 | 103.3 ± 3.2; 3 |
| *ADMRC154* |  | | F | H | CH_3_ | 93.9 ± 2.1; 3 | 64.7 ± 3.7; 4 | 110.3 ± 8.7 ; 12 |
| *ADMRC155* |  | | OCH_3_ | OCH_3_ | OCH_3_ | 104.0 ± 6.6; 4 | 89.3 ± 7.4; 3 | 97.5 ± 3.4; 3 |
| *ADMRC156* |  | | OH | OCH_3_ | OCH_3_ | 102.2 ± 8.8; 4 | 79.4 ± 4.6; 4 | 108.1 ± 6.5; 3 |
| *ADMRC157* |  | | OCH_3_ | OCH_3_ | Cl | 109.2 ± 1.5; 4 | 89.1 ± 2.8; 4 | 102.1 ± 0.01; 3 |
| *ADMRC158* |  | | OH | OCH_3_ | Cl | 97.4 ± 6.1; 4 | 85.3 ± 8.7; 4 | 119.1 ± 4.6; 5 (0.88) |
| *ADMRC159* |  | | H | H | Cl | 97.2 ± 7.8; 2 | 64.1 ± 2.7; 4 | 91.7 ± 6.5; 3 |
| *ADPKN160* |  | | OCH_3_ | H | OCH_3_ | 97.6 ± 3.7; 4 | 77.9 ± 3.6; 4 | 116.4 ± 4.2; 4 (0.87) |
| *ADPKN161* |  | | F | H | H | 90.8 ± 5.8; 4 | 83.5 ± 9.2; 3 | 126.7 ± 0.8; 3 (0.86) |
| *ADPKN 162* | OCH_3_ | OCH_3_ | OCH_3_ | H | OCH_3_ | 98.1 ± 4.7; 4 | 79.8 ± 3.4; 4 | 82.0 ± 1.6; 3 |
| *ADPKN163* | OCH_3_ | OCH_3_ | OCH_3_ | H | Cl | 113.6 ± 3.4; 4 | 82.3 ± 7.7; 3 | 88.0 ± 5.0; 4 |
| *ADPKN164* | OCH_3_ | OCH_3_ | F | H | Cl | 99.6 ± 5.7; 4 | 66.0 ± 3.4; 4 | 123.2 ± 7.0; 4 (0.83) |
| *ADPKN165* |  | | OCH_3_ | H | H | 107.1 ± 4.5; 4 | 83.5 ± 6.0; 4 | 166.3 ± 5.5; 7 (0.79) |

Enzyme activity is expressed as % TryS activity ± 2σ^n-1^ and for compounds that at 30 µM inhibit TryS by 45-55%, an estimated IC_50_ value of ~30 µM is provided. For compounds affecting BIOMOL GREEN signal, the interference factor used to correct TryS activity is provided in brackets (see Materials & Methods and S1 Text). The number of assay replicates is shown after the semicolon.
