# Supplementary material for: Identification of Novel Chemical Scaffolds Inhibiting Trypanothione Synthetase from Pathogenic Trypanosomatids
Source: PLoS Negl Trop Dis. 2016 Apr 12;10(4):e0004617. doi: 10.1371/journal.pntd.0004617 (PMC4829233; doi:10.1371/journal.pntd.0004617)
Supplement: S3 Table — (DOCX) [file pntd.0004617.s008.docx]

**Table S3. BDA, *N,N'*-bis(3,4-substituted-benzyl)DA.**

|  | | | | | | |
| --- | --- | --- | --- | --- | --- | --- |
|  | **Substitutions/ chain’s size** | | | **Activity ± 2σ^n-1^ (%); n**  **(interference factor)** | | |
| **Name** | **R_1_=R_4_** | **R_2_=R_3_** | **n** | ***Tc*TryS** | ***Li*TryS** | ***Tb*TryS** |
| *APC1-71B* | OBn | H | 3 | 99.0 ± 19.1; 2 | 80.8 ± 8.7; 3 | 66.0 ± 0.7; 3 |
| *APC1-79* | OBn | OCH_3_ | 3 | 63.3 ± 2.7; 2 | 72.1 ± 3.4; 4 (1.27) | 66.0 ± 2.9; 4 |
| *APC1-82* | OH | OCH_3_ | 3 | 92.9 ± 5.6; 4 | 84.9 ± 0.9; 4 | 88.1 ± 4.4; 3 |
| *APC1-67* | OCH_3_ | H | 3 | 92.4 ± 4.7; 4 | 102.2 ± 4.7; 4 | 71.9 ± 4.6; 4 |
| *APC1-87* | OBn | H | 4 | 73.4 ± 4.1; 2 | ~30 (1.26) | 62.1 ± 4.0; 4 |
| *APC1-89* | OBn | OCH_3_ | 4 | 74.4 ± 1.4; 2 | 76.6 ± 2.7; 4 (1.32) | ~30 (1.31) |
| *APC1-93* | OH | OCH_3_ | 4 | 94.4 ± 2.4; 4 | 101.3 ± 8.7. 4 | 94.0 ± 9.1; 5 |
| *APC1-99* | OBn | H | 6 | 66.6 ± 4.1;4 | 60.9 ± 3.4; 4 (1.33) | ~30 (1.05) |
| *APC1-101* | OBn | OCH_3_ | 6 | 83.0 ± 2.1; 3 (1.40) | 73.7 ± 10.1; 8 (1.36) | 38.8 ± 1.8; 3 (1.33) |
| *APC1-121* | OH | OCH_3_ | 6 | 92.9 ± 5.0; 4 | 95.0 ± 7.8; 4 | 91.1 ± 4.8; 3 |
| *APC1-98* | OCH_3_ | H | 6 | 94.2 ± 5.5; 2 | 79.6 ± 5.6; 4 | 72.8 ± 3.7; 4 |
| *EAP1-5 (I)* | H | H | 8 | 82.8 ± 5.2; 3 | 62.8 ± 3.9; 4 | 78.3 ± 3.6; 4 |
| *APC1-109* | OBn | H | 8 | 66.2 ± 10.9; 2 | 72.2 ± 6.2; 4 | 65.5 ± 1.2; 3 (1.12) |
| *APC1-111* | OBn | OCH_3_ | 8 | 71.1 ± 4.1; 3 (1.57) | ~30 (1.51) | 44.4 ± 3.2; 5 (1.52) |
| *APC1-122* | OH | OCH_3_ | 8 | 94.5 ± 7.0; 4 | 102.8 ± 8.6; 3 | 91.6 ± 3.6; 3 |
| *EAP1-45* | CH_3_ | H | 8 | 92.3 ± 4.5; 4 | 91.9 ± 2.7; 3 | 78.4 ± 0.6; 3 |
| *EAP1-37* | iPr | H | 8 | 65.6 ± 5.3; 4 (1.46) | 77.4 ± 7.9; 9 (1.284) | 56.8 ± 5.6; 3 (1.43) |
| *EAP1-39* | F | H | 8 | 96.0 ± 4.0; 4 | 69.6 ± 5.5; 4 | 85.7 ± 2.0; 3 |
| *EAP1-43* | Cl | H | 8 | 87.9 ± 4.7; 4 | 78.0 ± 5.9; 4 | 69.7 ± 3.0; 4 |
| *EAP1-46* | Br | H | 8 | 93.7 ± 7.6; 3 | 61.8 ± 3.9; 3 | 73.4 ± 1.7; 3 |
| *APC1-113* | H | H | 10 | 88.5 ± 6.6; 3 | 86.9 ± 6.3; 3 | 89.5 ± 3.6; 3 |
| *APC1-117* | OBn | H | 10 | 90.8 ± 12.3; 2 | 69.8 ± 6.5; 3 | 71.4 ± 6.1; 4 |
| *APC1-119* | OBn | OCH_3_ | 10 | 101.5 ± 1.9; 4 (1.18) | 78.1 ± 9.8; 6 (1.219) | 55.5 ± 1.8. 3 (1.11) |
| *APC1-123* | OH | OCH_3_ | 10 | 88.8 ± 6.5; 4 | 94.3 ± 8.2; 3 | 86.0 ± 5.5; 3 |
| *APC1-115* | OCH_3_ | H | 10 | 76.1 ± 2.5; 4 | 79.8 ± 2.3; 3 | 69.0 ± 6.3; 3 |
| *EAP1-55* | CH_3_ | H | 10 | 68.6 ± 3.9; 2 | 71.5 ± 5.8; 4 | 61.8 ± 4.8; 3 |
| *EAP1-47* | iPr | H | 10 | ~30 (1.48) | ~30 (1.34) | ~30 (1.49) |
| *EAP1-51* | F | H | 10 | 87.5 ± 4.6; 4 | 80.6 ± 2.7; 4 | 81.0 ± 7.5; 3 |
| *EAP1-53* | Cl | H | 10 | 73.3 ± 2.3; 4 | 69 ± 5.7; 4 (1.305) | 63.9 ± 4.1; 4 (1.34) |
| *EAP1-57* | Br | H | 10 | 81.2 ± 4.2; 3 | 75.2 ± 4.5; 3 | 62.6 ± 3.6; 3 (1.26) |
| *EAP1-9* | H | H | 12 | 87.3 ± 5.4; 4 | 89.6 ± 9.4; 3 | 74.9 ± 4.1; 4 |
| *EAP1-11* | OCH_3_ | H | 12 | 82.8 ± 7.6; 3 | 62.0 ± 7.8; 4 | 77.2 ± 4.5; 5 (1.30) |
| *EAP1-69* | F | H | 12 | 56.3 ± 3.6; 3 | 63.2 ± 5.0; 4 | 81.4 ± 5.6; 4 (1.39) |
| *EAP1-67* | Cl | H | 12 | 76.2 ± 6.6; 4 (1.34) | ~30 (1.35) | 57.6 ± 1.6; 4 (1.33) |
| *EAP1-63* | Br | H | 12 | 69.2 ± 3.8; 4 (1.64) | 74.1 ± 9.3; 6 | ~30 (1.30) |

Enzyme activity is expressed as % TryS activity ± 2σ^n-1^ and for compounds that at 30 µM inhibit TryS by 45-55%, an estimated IC_50_ value of ~30 µM is provided. For compounds affecting BIOMOL GREEN signal, the interference factor used to correct TryS activity is provided in brackets (see Materials & Methods and S1 Text). The number of assay replicates is shown after the semicolon.
