# Supplementary material for: Identification of Novel Chemical Scaffolds Inhibiting Trypanothione Synthetase from Pathogenic Trypanosomatids
Source: PLoS Negl Trop Dis. 2016 Apr 12;10(4):e0004617. doi: 10.1371/journal.pntd.0004617 (PMC4829233; doi:10.1371/journal.pntd.0004617)
Supplement: S4 Table — (DOCX) [file pntd.0004617.s009.docx]

**Table S4. BZ, benzofuroxan derivatives.**

|  | | | | | | | | | | |
| --- | --- | --- | --- | --- | --- | --- | --- | --- | --- | --- |
|  | **Substitutions** | | | | | | **Activity ±**  **2σ ^n-1^ (%); n**  **(interference factor)** | | | |
| **Name** | **R_1_** | | **R_2_** | **R_3_** | | **R_4_** | ***Tc*TryS** | ***Li*TryS** | | ***Tb*TryS** |
| *J09/J34* | H | | H | H | | H | 102.2 ± 3.3; 3 | 84.9 ± 5.5; 4 | | 90.9 ± 5.9; 4 |
| *J10* | H | | CH_3_ | H | | H | 92.6 ± 10.8; 3 | 84.7 ± 10.5; 4 | | 92.7 ± 3.4; 3 |
| *J11* | H | | CH_3_ | CH_3_ | | H | 86.4 ± 2.9; 4 | 103.6 ± 10.4; 3 | | 88.8 ± 5.8; 4 |
| *J12* | H | | Cl | H | | H | 63.7 ± 0.5; 4 | 93.8 ± 3.9; 4 | | 92.7 ± 2.7; 3 |
| *J27* | H | | I | H | | H | 90.1 ± 5.9; 3 | 71.0 ± 8.0; 3 | | 98.2 ± 5.6; 3 |
| *J31* | H | | F | H | | H | 118.8 ± 6.1; 4 (1.00) | 84.1 ± 0.001; 3 | | 96.2 ± 3.7; 4 |
| *J40* | H | | F | F | | H | 87.4 ± 1.6; 3 | 92.9 ± 5.1; 4 | | 85.0 ± 1.4; 2 |
| *J33* | H | | Br | H | | H | 98.1 ± 4.0; 3 | 76.0 ± 6.8; 3 | | 96.5 ± 6.3; 3 |
| *J13* | H | | Cl | Cl | | H | 84.9 ± 4.4; 3 | 102.2 ± 7.8; 3 | | 88.9 ± 2.7; 3 |
| *J26* | Cl | | H | Cl | | H | 95.8 ± 1.1; 3 | 88.0 ± 1.0; 3 | | 104.9 ± 5.7; 3 |
| *J14* | H | | OCH_3_ | H | | H | 77.2 ± 4.4; 4 | 96.1 ± 7.3; 4 | | 93.1 ± 5.6; 4 |
| *J32* | H | | OCH_3_ | OCH_3_ | | H | 98.1 ± 6.4; 4 | 72.7 ± 7.2; 4 | | 93.4 ± 4.5; 4 |
| *J16* | H | | N_2_O | H | | H | 76.5 ± 1.0; 3 | 80.1 ± 9.2; 4 | | 92.6 ± 4.9; 3 |
| *J38* | N_2_O | | H | H | | H | 90.6 ± 9.5; 3 | 88.4 ± 4.2; 3 | | 91.1 ± 0.7; 2 |
| *J17* | N_2_O | | H | N_2_O | | H | 73.2 ± 13.1; 3 | 96.3 ± 1.5; 5 | | 89.2 ± 6.4; 3 |
| *J25* | H | | CF_3_ | H | | H | 81.8 ± 2.2; 3 | 110.7 ± 2.4; 3 | | 98.4 ± 4.2; 3 |
| *J15* | H | |  | | | H | 76.7 ± 15.2; 3 | 97.9 ± 7.4; 3 | | 88.4 ± 8.2; 4 |
| *J37* | H | |  | | | H | 97.0 ± 2.4; 3 | 98.2 ± 3.3; 3 | | 86.3 ± 2.2; 3 |
| *J36* | H | | N_2_O | | N_2_O | H | 95.9 ± 1.4; 2 | 66.8 ± 3.2; 4 | | 106.1 ± 2.5; 3 |
| *J18* | H | |  | | | H | ~30 (1.00) | 58.7 ± 3.6; 4 (1.00) | | 92.7 ± 5.9; 3 |
| *J19* | H | |  | | | H | 73.5 ± 5.9; 4 | 104.0 ± 11.7; 6 (0.99) | | 99.6 ± 5.8; 3 |
| *J20* | |  | |  | | | 64.4 ± 3.8; 4 | 111.5 ± 14.5; 12 (0.97) | 118.8 ± 2.4; 3 (0.97) | |
| *J21* | | H | H |  | | | 62.4 ± 1.7; 3 | 71.7 ± 4.4; 4 | 112.2 ± 4.8; 3 (0.97) | |
| *J23* | | H |  | | | H | 96.7 ± 2.0; 4 | 94.5 ± 1.4; 3 | 108.4 ± 0.9; 3 | |
| *J24* | | H |  | | | H | 84.7 ± 5.7; 4 | 105.3 ± 8.5; 4 | 102.7 ± 7.2; 3 | |

Enzyme activity is expressed as % TryS activity ± 2σ^n-1^ and for compounds that at 30 µM inhibit TryS by 45-55%, an estimated IC_50_ value of ~30 µM is provided. For compounds affecting BIOMOL GREEN signal, the interference factor used to correct TryS activity is provided in brackets (see Materials & Methods and S1 Text). The number of assay replicates is shown after the semicolon.
