# Supplementary material for: Identification of Novel Chemical Scaffolds Inhibiting Trypanothione Synthetase from Pathogenic Trypanosomatids
Source: PLoS Negl Trop Dis. 2016 Apr 12;10(4):e0004617. doi: 10.1371/journal.pntd.0004617 (PMC4829233; doi:10.1371/journal.pntd.0004617)
Supplement: S5 Table — (DOCX) [file pntd.0004617.s010.docx]

**Table S5. AI (AP), 4,5-dihydroazepino[4,5-*b*]indol-2(1*H*,3*H*,6*H*)-one derivatives, 4-azapaullones derivatives.**

|  | | | | |
| --- | --- | --- | --- | --- |
|  |  | **Activity ± 2σ ^n-1^ (%); n** | | |
| **Name** | **–Substitution** | ***Tc*TryS** | ***Li*TryS** | ***Tb*TryS** |
| ***4a*** | Ph - | 101.5 ± 1.9; 3 | 75.5 ± 3.5; 2 | 61.9 ± 6.4; 4 |
| ***4b*** | 4-MeO-Ph- | 98.1 ± 3.2; 4 | 55.9 ± 2.6; 3 | 77.6 ± 5.4; 4 |
| ***4c*** | 4-Cl-Ph- | 106.3 ± 5.8; 3 | 68.0 ± 5.0; 4 | 79.8 ± 5.7; 4 |
| ***4d*** | Furan-2-yl- | 103.7 ± 5.9; 4 | 63.3 ± 5.0; 4 | 90.2 ± 3.8; 4 |
| ***4e*** | (CH_3_CH_2_)_2_N- | 90.0 ± 4.7; 4 | 84.4 ± 6.3; 3 | 91.5 ± 5.2; 4 |
| ***4f*** | PhNH- | 83.4 ± 5.9; 3 | 71.0 ± 4.3; 3 | 68.1 ± 2.4; 4 |
| ***4g*** | PhCH_2_NH- | 84.3 ± 4.6; 4 | 70.6 ± 7.8; 3 | 67,5 ± 4,2; 6 |
|  | | | | |
| **Name** | **Substitution** | ***Tc*TryS** | ***Li*TryS** | ***Tb*TryS** |
| ***5a*** | Ph - | 105.7 ± 4.1; 3 | 72.4 ± 6.0; 3 | 91.1 ± 7.2; 3 |
| ***5b*** | 4-MeO-Ph- | 102.2 ± 4.4; 3 | 69.4 ± 5.3; 4 | 80.4 ± 3.1; 3 |
| ***5c*** | 4-Cl-Ph- | 94.6 ± 5.9; 4 | 64.9 ± 2.1; 2 | 73.2 ± 7.8; 4 |
| ***5d*** | (CH_3_CH_2_)_2_N- | 99.0 ± 2.0; 4 | 94.5 ± 5.6; 3 | 102.0 ± 8.3; 4 |
| ***5e*** | PhNH- | 84.2 ± 5.7; 4 | 91.3 ± 2.0; 3 | 94.6 ± 2.7; 4 |
| ***5f*** | PhCH_2_NH- | 93.4 ± 3,5; 4 | 81.3 ± 4.0; 2 | 81.8 ± 5.5; 4 |
|  | | | | |
| **Name** | **–Substitutions** | ***Tc*TryS** | ***Li*TryS** | ***Tb*TryS** |
| ***11a*** | R_1_ = I, R_2_ = H | 106.3 ± 4.3; 4 | 61,3 ± 5,0; 2 | 96.7 ± 6.1; 4 |
| ***11b*** | R_1_ = H, R_2_ = I | 97.6 ± 6.0; 3 | 73,8 ± 0,9; 4 | 111.7 ± 8.1; 3 |

Enzyme activity is expressed as % TryS activity ± 2σ^n-1^ and for compounds that at 30 µM inhibit TryS by 45-55%, an estimated IC_50_ value of ~30 µM is provided. For compounds affecting BIOMOL GREEN signal, the interference factor used to correct TryS activity is provided in brackets (see Materials & Methods and S1 Text). The number of assay replicates is shown after the semicolon.
