# Supplementary material for: Identification of Novel Chemical Scaffolds Inhibiting Trypanothione Synthetase from Pathogenic Trypanosomatids
Source: PLoS Negl Trop Dis. 2016 Apr 12;10(4):e0004617. doi: 10.1371/journal.pntd.0004617 (PMC4829233; doi:10.1371/journal.pntd.0004617)
Supplement: S6 Table — (DOCX) [file pntd.0004617.s011.docx]

**Table S6. PD, 1*H*-purine-2,6(3*H*,7*H*)-dione derivatives.**

|  | | | | | | | |
| --- | --- | --- | --- | --- | --- | --- | --- |
|  | **Substitutions** | | | | **Activity ±**  **2σ ^n-1^ (%); n**  **(interference factor)** | | |
| **Name** | **R_1_** | **R_2_** | **R_3_** | **R_4_** | ***Tc*TryS** | ***Li*TryS** | ***Tb*TryS** |
| *TC226* | CH_3_ | CH_3_ |  | H_2_N (CH_2_)_2_ O(CH_2_)_2_  OH | 98.3 ± 1.3; 2 | 79.2 ± 7.7; 4 | 83.9 ± 7.1; 6 |
| *TC227* | CH_3_ | CH_3_ |  |  | 161.6 ± 5.3; 3  (0.98) | 88.5 ± 7.1; 4 | 102.0 ± 2.3; 3 |
| *C3* | H | CH_3_(CH_2_)_3_ | CH_3_ |  | 91.1 ± 4.6; 4 | 84.5 ± 3.3; 3 | 92.7 ± 3.6; 3 |

Enzyme activity is expressed as % TryS activity ± 2σ^n-1^ and for compounds that at 30 µM inhibit TryS by 45-55%, an estimated IC_50_ value of ~30 µM is provided. For compounds affecting BIOMOL GREEN signal, the interference factor used to correct TryS activity is provided in brackets (see Materials & Methods and S1 Text). The number of assay replicates is shown after the semicolon.
