# Supplementary material for: Identification of Novel Chemical Scaffolds Inhibiting Trypanothione Synthetase from Pathogenic Trypanosomatids
Source: PLoS Negl Trop Dis. 2016 Apr 12;10(4):e0004617. doi: 10.1371/journal.pntd.0004617 (PMC4829233; doi:10.1371/journal.pntd.0004617)
Supplement: S7 Table — (DOCX) [file pntd.0004617.s012.docx]

**Table S7. AOCA, 2-aminooxazole-5-carboxamide derivatives.**

|  | | | | | |
| --- | --- | --- | --- | --- | --- |
|  | **Substitutions** | | **Activity ± 2σ ^n-1^ (%); n** | | |
| **Name** | **R_1_** | **R_2_** | ***Tc*TryS** | ***Li*TryS** | ***Tb*TryS** |
| *ZVR102* | OBn | OBn | 87.1 ± 4.7; 3 | 95.3 ± 6.7; 4 | 97.3 ± 5.6; 3 |
| *ZVR103* | Bu | Bu | 100.0 ± 5.0; 2 | 99.4 ± 5.6; 4 | 82.4 ± 4.7; 3 |
| *ZVR113* | OBn | Bu | 82.9 ± 3.5; 4 | 93.6 ± 7.1; 3 | 99.6 ± 6.2; 3 |
| *NOTE:* *OBn,*  *means O-benzyl substitution. Bu means Butyl substitution.* | | | | | |

Enzyme activity is expressed as % TryS activity ± 2σ^n-1^ and for compounds that at 30 µM inhibit TryS by 45-55%, an estimated IC_50_ value of ~30 µM is provided. For compounds affecting BIOMOL GREEN signal, the interference factor used to correct TryS activity is provided in brackets (see Materials & Methods and S1 Text). The number of assay replicates is shown after the semicolon. OBn and Bu indicate O-benzyl and butyl substitutions, respectively.
