# Supplementary material for: Identification of Novel Chemical Scaffolds Inhibiting Trypanothione Synthetase from Pathogenic Trypanosomatids
Source: PLoS Negl Trop Dis. 2016 Apr 12;10(4):e0004617. doi: 10.1371/journal.pntd.0004617 (PMC4829233; doi:10.1371/journal.pntd.0004617)
Supplement: S8 Table — (DOCX) [file pntd.0004617.s013.docx]

**Table S8. Kinetic characterization conditions for recombinant TryS.**

| *Tc*TryS Silvio X10 clone 7 [25]: PK/LDH assay, at 25ºC, using buffer 100 mM HEPPS-K^+^, pH 8.0, 0.2 mM NADH, 1 mM PEP, 5 mM DTT, 0.5 mM EDTA, 10 mM MgSO_4_, 2 units.mL^-1^ PK and LDH, 0.8 µM *Tc*TryS. Substrates saturating conditions: 2 mM ATP and 10 mM SP. GSH 1 mM. |
| --- |
| ***Tc*TryS Tulahuen 0: PK/LDH assay, at 25ºC, using buffer 100 mM HEPES-K^+^, pH 7.4, 0.2 mM NADH, 1 mM PEP, 5 mM DTT, 0.5 mM EDTA, 10 mM MgSO_4_, 1.5 units PK and LDH, ~2.5 x 10^-6^ µmol.min^-1^.mL^-1^ µM enzyme. Substrates saturating conditions: 5 mM ATP, 18 mM SP and 0.57 mM GSH.** |
| *Tc*TryS Ninoa [22]: PK/LDH assay, at 37ºC, using buffer 40 mM HEPES-NaOH, pH 7.4, 0.2 mM NADH, 1 mM PEP, 5 mM DTT, 1 mM EDTA, 5 mM MgCl_2_, 2-2.8 units PK and LDH, 2-5 µg enzyme. Substrates saturating conditions: 0.7 mM ATP, GSH 7.6 mM and 11 mM SP. |
| *Tb*TryS 427 (MITat1.4) [27]: PK/LDH assay, at 25ºC, using buffer 100 mM HEPPS-K^+^, pH 8.0, 0.2 mM NADH, 1 mM PEP, 5 mM DTT, 0.5 mM EDTA, 10 mM MgSO_4_, 2 units.mL^-1^ PK and LDH, 1 µM enzyme. Substrates saturating conditions: 2 mM ATP and 10 mM SP. GSH 0.1 mM. |
| *Tb*TryS 427 (MITat1.4) [19]: end-point assay using BIOMOL GREEN ^TM^ reagent, at RT, using buffer 100 mM HEPES-K^+^, pH 8.0, 2 mM DTT, 0.01% Brij-35, 0.5 mM EDTA, 10 mM magnesium acetate, 10 nM enzyme. Substrates saturating conditions: 0.1 mM ATP and 1.2 mM SP. GSH 0.1 mM. |
| ***Tb*TryS (MITat1.4): PK/LDH assay, at RT (20-25ºC), using buffer 100 mM HEPES-K^+^, pH 7.4, 5 mM DTT, 0.5 mM EDTA, 10 mM MgSO_4_, ~10 x 10^-6^ µmol.min^-1^.mL^-1^ enzyme. Substrates saturating conditions: 5 mM ATP and 9 mM SP and 0.33 mM GSH.** |
| *Tb*TryS 427 [24]: PK/LDH assay, at 25ºC, using buffer 100 mM HEPES-K^+^, pH 8.0, 0.2 mM NADH, 1 mM PEP, 5 mM DTT, 0.5 mM EDTA, 10 mM MgCl_2_, 2 units PK and LDH, 0.2-0.8 µM enzyme. Substrates saturating conditions: 2.1 mM ATP and 20 mM SP. GSH 0.1 mM. |
| *Lm*TryS Friedlin [26]: PK/LDH assay, at 25ºC, using buffer 50 mM HEPPS-K^+^, 5 mM TCEP, pH 7.7, 0.2 mM NADH, 1 mM PEP, 5 mM DTT, 0.5 mM EDTA, 10 mM MgSO_4_, 2 units PK and LDH, 0.4 µM enzyme. Substrates saturating conditions: 2 mM ATP and 10 mM SP. GSH 0.25 mM. |
| ***Li*TryS JPCM5. PK/LDH assay, at 25ºC, using buffer 100 mM HEPES-K^+^, pH 7.4, 0.2 mM NADH, 1 mM PEP, 5 mM DTT, 0.5 mM EDTA, 10 mM MgSO_4_, 1.5 units PK and LDH, ~2.3 x 10^-6^ µmol.min^-1^.mL^-1^ enzyme. Substrates saturating conditions: 5 mM ATP, 18 mM SP and 0.25 mM GSH.** |
| *Cf*TryS [23]: PK/LDH assay, at 25ºC, using buffer 100 mM HEPES-K^+^, pH 7.2, 0.2 mM NADH, 1 mM PEP, 5 mM DTT, 0.5 mM EDTA, 10 mM MgSO_4_, 2 units.mL^-1^ PK and LDH, 0.25 µM enzyme. Substrates saturating conditions: 2.5 mM ATP and 10 mM SP. GSH 1 mM. |

**The conditions employed in this work to perform the kinetic characterization of tritryp TryS are shown in bold letters.**
