# Supplementary material for: Identification of Novel Chemical Scaffolds Inhibiting Trypanothione Synthetase from Pathogenic Trypanosomatids
Source: PLoS Negl Trop Dis. 2016 Apr 12;10(4):e0004617. doi: 10.1371/journal.pntd.0004617 (PMC4829233; doi:10.1371/journal.pntd.0004617)
Supplement: S9 Table — (DOCX) [file pntd.0004617.s014.docx]

**Table S9. SP-competitive inhibition of *Li*TryS by MOL2008**

| **SP (mM)** | **% TryS inhibition** |
| --- | --- |
| 16 | 31 ± 2 |
| 8 | 36 ± 3 |
| 4 | 44 ± 5 |
| 2 | 50 ± 4 |
| 1 | 59 ± 4 |
| 0.2 | 90 ± 6 |

The assay was performed as indicated in Materials and Methods and S1 Text (End-point assay with BIOMOL Green reagent). The concentration of GSH, ATP and inhibitor (MOL2008) was fixed at 250 uM, 150 uM and 150 nM (IC_50_ value) whereas SP concentration was varied in the range 0.2 to 16 mM. The data correspond to n=4 experimental determinations and the activity is referred as percentage inhibition ± 2σ^n-1^ relative to a control reaction at the corresponding SP concentration (with fixed cocentration of co-substrates) and in the absence of inhibitor but with 10% v/v DMSO (compound vehicle).
