# Supplementary material for: Identification of Novel Chemical Scaffolds Inhibiting Trypanothione Synthetase from Pathogenic Trypanosomatids
Source: PLoS Negl Trop Dis. 2016 Apr 12;10(4):e0004617. doi: 10.1371/journal.pntd.0004617 (PMC4829233; doi:10.1371/journal.pntd.0004617)
Supplement: S10 Table — (DOCX) [file pntd.0004617.s015.docx]

**Table S10. Characterization of compounds enhancing tritryp trypanothione synthetase (TryS) activity.**

|  | | | % TryS activity ^a^ | | | |
| --- | --- | --- | --- | --- | --- | --- |
| Compound | Core structure | TryS ^b^ | ATP/GSH/SP | ATP/GSH | ATP/SP | ATP |
| none | - | *Tc*  *Tb* | 100.0 ± 3.0; 4  100.0 ± 2.3; 4 | 0.7 ± 3.1; 4 0.2 ± 3.0; 3 | 1.1 ± 3.1; 3  0.1 ± 2.0; 4 | 0.4 ± 3.2; 4 0.2 ± 2.0; 3 |
| ZVR159 | APPDA | *Tc* | 168.6 ± 4.1; 4  (1.10) | 1.2 ± 3.1; 3 | 0.2 ± 4.0; 4 | 1.2 ± 5.1; 4 |
| TC227 | PD | *Tc* | 161.6 ± 5.3; 3  (0.98) | 1.1 ± 3.1; 4 | 0.2 ± 3.1; 4 | 1.1 ± 2.1; 4 |
| ADPKN165 | BBHPP | *Tb* | 166.3 ± 5.5; 7  (0.79) | 1.1 ± 5.2; 4 | 0.3 ± 2.3; 4 | 3.2 ± 5.2; 3 |

The enzymatic assays were performed as indicated in Materials and Methods and S1 Text (End-point assay with BIOMOL Green reagent) for *Tb*TryS and *Tc*TryS.

^a^ Percentage TryS activity for the different enzymes and substrate combinations with or whithout compounds is calculated relative to control reactions for the respective TryS in the presence of all substrates (ATP/GSH/SP) and 10% DMSO (compound solvent). These controls are indicated with 100% values in the table.

^b^ *Tc*, *Trypanosoma cruzi*; *Tb*, *Trypanosoma brucei brucei*.
