# Supplementary material for: Identification of Novel Chemical Scaffolds Inhibiting Trypanothione Synthetase from Pathogenic Trypanosomatids
Source: PLoS Negl Trop Dis. 2016 Apr 12;10(4):e0004617. doi: 10.1371/journal.pntd.0004617 (PMC4829233; doi:10.1371/journal.pntd.0004617)
Supplement: S11 Table — (DOCX) [file pntd.0004617.s016.docx]

**Table S11. Analysis of intracellular thiols in bloodstream *T. b. brucei* and *L. infantum* promastigotes**

|  | ***T. b. brucei* TryS-RNAi cell line** | | ***T. b. brucei* WT** | | ***L. infantum* WT^a^** | |
| --- | --- | --- | --- | --- | --- | --- |
| nmol thiol x 5x10^7^ cells^-1^ | non-induced | induced ^b^ | non-treated | EAP1-47  [100 nM] ^b^ | non-treated | MOL2008  [12 µM] ^b^ |
| T(SH)_2_ | 2.8 | ≤ 1  (≤ 36%) | 6.2 | 4.5  (72%) | 37.4  13.0 | 3.7 (10%)  ≤ 1 (≤ 8%) |
| GSH | 2.5 | 5.8  (232%) | 5.9 | 8.2  (139%) | 4.4  10.0 | < 0.5 (≤ 11%)  2.9 (29%) |

^a^ The values from two independent experiments are shown.

^b^ Values in brackets refer to the content of the corresponding thiol expressed as percentage relative to the level of this species in non-treated cells.
