# Supplementary material for: Identification of Novel Chemical Scaffolds Inhibiting Trypanothione Synthetase from Pathogenic Trypanosomatids
Source: PLoS Negl Trop Dis. 2016 Apr 12;10(4):e0004617. doi: 10.1371/journal.pntd.0004617 (PMC4829233; doi:10.1371/journal.pntd.0004617)
Supplement: S1 Text — (DOCX) [file pntd.0004617.s017.docx]

**Supplemental Text 1**

**Heterologous Expression and Purification of Recombinant TryS.** Starter cultures for each host/expression system were prepared with freshly transformed cells inoculated in LB medium with the corresponding antibiotics for selection and grown overnight under aerobic conditions at 37 °C and 180 rpm.

For *Tb*TryS, cells were inoculated 1:100 in Terrific Broth medium supplemented with 10 g/L glucose and 100 μg/mL ampicillin and grown at 37 °C and 180 rpm until an optical density of ~0.8-1.0 at 600 nm. The cultures were then chilled at 4 °C for 15 min and expression of recombinant protein was induced with 0.5 mM isopropyl 1-thio-β-D-galactopyranoside (IPTG) for 5 h at 25 °C and 180 rpm. For *Tc*TryS and *Li*TryS, cells were inoculated 1:100 in ZYM-5052 auto-induction medium lacking the trace elements supplement [1] and containing 100 μg/mL ampicillin or 50 μg/mL kanamycin, respectively. After grow at 37 °C and 180 rpm for 5 h, the cultures were incubated at 25 °C and 120 rpm for further 16-18 h.

Cells harvested by centrifugation at 4,000*g* for 15 min at 4 °C (Sorvall RC-6, Thermo Fisher Scientific) were resuspended at a ratio of 1 g wet weight pellet per 5 ml buffer A (50 mM NaH_2_PO_4_ pH 7.2, 500 mM NaCl) with 10 mM imidazole. Cell lysis was achieved by gently shaking with lysozyme (30 mg % w/v) for 1 h at 4 °C followed by sonication on ice (4 pulses during 30 seconds interspersed by pauses of 60 seconds) at amplitude of 40 % using a Digital Sonifier 450 (Branson). Ten mM MgSO_4_ and 2 U/mL DNase were added 30 min after the lysis was initiated. The lysate was centrifuged twice at 20,000*g* for 20 min at 4 °C to remove debris and the supernatant was cleared by filtration (0.45 μM filter) and then loaded onto two 1 mL HisTrap Fast Flow columns (GE Healthcare) connected in tandem and pre-equilibrated in buffer A with 10 mM imidazole. The columns were washed with buffer A supplemented with 25 mM imidazole and the recombinant protein was eluted in an isocratic fashion with 10 mL of buffer B (buffer A with 500 mM imidazole). This chromatography was performed at 4 ºC and at a flow rate of 1 mL/min using a peristaltic pump (TRIS Teledyne ISCO). Fractions containing active TryS (tested by the lactate dehydrogenase (LDH)/pyruvate kinase (PK) assay, see “*Kinetic characterization”*) were pooled and subjected to diafiltration with 50 mM Tris, pH 7.4 with a 30 kDa cut-off Amicon filter (Millipore) and repeated centrifugation at 4,000*g* at 4 ºC (Thermo-Sorvall centrifuge). The diafiltrated samples were loaded onto a MonoQ HR 5/5 column (GE Healthcare) and the proteins eluted with a linear gradient from 0 to 1 M NaCl. Peak fractions containing active TryS were tested by the end point assay (see below “*TryS screening assay development”*), pooled and concentrated with a 30 kDa cut-off Amicon filter (Millipore). The final purification step consisted on a size exclusion chromatography (SEC) with a Superdex^TM^ 200 10/300 GL column (GE Healthcare) equilibrated in reaction buffer (see below *“Kinetic characterization”*) containing 150 mM NaCl and run at 0.75 mL/min. All TryS eluted with a retention volume compatible with a monomeric species of about 74-78 kDa. The elution fractions containing recombinant TryS were pooled, concentrated to ≥ 1 mg protein/mL using a 30 kDa cut-off Amicon filter (Millipore), supplemented with glycerol (final concentration of 40 % v/v) and stored at - 20 °C. Under these conditions the enzymes retained fully activity for at least 6 months.

The last two chromatographies were performed at room temperature (RT, 20-25 ºC) using an Äkta-FPLC device (GE Healthcare). After the final chromatography step, enzyme purity and activity were assessed by SDS-PAGE (12 %) under reducing conditions and the end-point TryS assay (see below “*TryS screening assay development”*), respectively.

**Kinetic Characterization.**

The kinetic characterization of His-tagged TryS was performed using the LDH/PK assay which couples ATP regeneration to NADH oxidation. All reactions were performed at RT in 100 mM HEPES-K, 0.5 mM EDTA, 10 mM MgSO_4_, pH 7.4 (reaction buffer). Ammonium sulfate suspensions of PK and LDH were dissolved in reaction buffer at 1000 U/mL immediately prior to use. In a final volume of 0.15 ml (quarz cuvette), each reaction contained 0.2 mM NADH, 1 mM PEP, 5 mM DTT, 10 U/mL PK, 10 U/mL LDH and 0.86 µM *Tc*TryS or 2.95 µM *Tb*TryS or 0.81 µM *Li*TryS, and varying concentrations of ATP (0 to 5 mM), GSH (4.1 µM to 18 mM) or SP (0 and 18 mM) at fixed saturating concentrations of the corresponding cosubstrates (5 mM ATP, 18 mM SP and 0.57, 0.33 and 0.25 mM GSH for *Tc*TryS, *Tb*TryS and *Li*TryS, respectively). The reactions samples were preincubated for 3 min at RT and started by the addition of GSH containing 5 mM DTT (for determination of GSH *K*_M_ and *K_i_*) or SP (for determination of ATP and SP *K*_M_). Absorbance at 340 nm was monitored with a Varian Cary 50 Bio UV-Visible spectrophotometer. The consistency of the dataset was validated by the smoothness of the plots and the reproducibility of replicates (n= 3-5) from at least 2 points of the curve (substrate concentrations corresponding to maximum and medium velocity). Outliers from the replicates were eliminated applying the Grubb´s test and plot fitting consistency with r-square values ≥0.96.

The end-point assay based on detection of inorganic phosphate (Pi) by the BIOMOL GREEN reagent was used to estimate the apparent *K_i_* for ADP. The reaction was performed in a total volume of 50 µL/well using 96-well plates. For all TryS, SP concentration was fixed at 2 mM and ADP was varied from 0 to 300 µM. GSH was adjusted to 0.05, 0.57 and 0.25 mM for *Tb*TryS, *Tc*TryS and *Li*TryS. The concentrations of ATP that were tested are 37.5, 75 and 150 µM for *Tb*TryS, 100, 150 and 400 µM for *Tc*TryS, and 100, 200 and 400 µM for *Li*TryS. The reaction was initiated by adding 5 µL of TryS (10, 2.5 and 2.3 x 10^-6^ µmol.min^-1^.mL^-1^ for *Tb*TryS, *Tc*TryS and *Li*TryS, respectively) or 5 µL reaction buffer containing 150 mM NaCl, for the blank, and stopped after 15 min with 200 µL BIOMOL GREEN reagent. The colorimetric signal was allowed to develop for 20 min and then absorbance at 650 nm (A_650_) was measured with a MultiScan EX plate reader (Thermo SCIENTIFIC).

The apparent kinetic parameters (*K_M_* and V_max_) were calculated by fitting plots of initial velocity (*v*) *vs.* substrate concentracion ([S]), determined at saturating concentration of co-substrates, to the Michaelis Menten equation assisted by the software OriginPro 8. For GSH, the *K_M_* and *K_i_* values were determined using the following equation *v* =*V_max_* / (1 + *K_M_* / [GSH] + [GSH] / *K_i_*), which considers the nonproductive binding of GSH to the substituted enzyme [2]. The apparent *K_i_* for ADP was estimated from linear fitting of the plots [E]/*v* *vs*. [ADP] at different concentrations of ATP, where [E] is enzyme concentration.

**Compound Interference**

For compounds that interfere with the colorimetric reaction, the corresponding interference factor (F) was determined as follow. Per well, 40 µL MM containing 25 µM K_2_HPO_4_ is added of 5 µL compound at different concentrations (e.g. 300 µM for primary screening or the concentration range for IC_50_ determinations) or 5 µL DMSO as reference and 5 µL screening reaction buffer with 150 mM NaCl. After gentle mixing, 200 µL BIOMOL GREEN reagent is added per well, the microtiter plate is incubated 20 min at RT and then A_650 nm_ is measured. F is calculated as: *F =* 1 + [1-(*A_650nm Ci_* / *A_650n DMSO_*)], where *A_650nm Ci_* is the mean absorbance at 650 nm of the compound *i* at 30 µM and *A_650nm DMSO_* is the mean absorbance at 650 nm of the reference. F values above or below 1 denote interference and are used to correct the relative % of TryS activity.

**Cell Culture and Cytotoxicity Assays**

Cell lines from bloodstream *T. b. brucei* (strain 427, cell line 514-1313 and 514-1313_RNAi-TryS) were cultivated aerobically in a humidified incubator at 37 °C with 5% CO_2_ in HMI-9 medium [3] supplemented with 10% (v/v) fetal bovine serum (FBS), 10 U/mL penicillin, 10 µg/mL streptomycin. The growth medium for WT and RNAi-TryS parasites was additionally supplemented with 0.2 µg/mL phleomycin and 2.5 µg/mL G418, and 5 µg/mL hygromycin only for the RNAi-TryS cell line. The RNAi of TryS was induced by adding 10 µg/mL oxytetracycline to the culture medium. The test compounds were dissolved and diluted in DMSO to obtain 3-24 mM stock solutions and the corresponding experimental concentrations to assess their EC_50_. Controls included 1% (v/v) DMSO and 5 µM Nifurtimox (Lampit from Bayer). Parasite viability was assessed in samples from cultures induced 48 h or not with oxytetracycline and grown in the presence and absence of compounds for 24 h, by manual cell counting (24-well culture plate) [4] or flow cytometry (96-well culture plate) [5].

*Leishmania infantum* promastigotes were cultured at 28ºC in RPMI 1640 Glutamax supplemented with 10% (v/v) FCS, 10 U ml^-1^ penicillin, 10 µg ml^-1^ streptomycin and 25 mM HEPES sodium salt pH 7.4. Parasites from synchronized cultures were seeded at 5×10^5^ cells/mL in 96-well plates in complete RPMI medium with varying concentrations (0.2-5 µM) of compounds in 1% (v/v) DMSO. Twenty four hours later the number of living parasites was estimated by cell counting under the light microscope using a Neubauer chamber and viability calculated as percentage relative to cultures incubated with the vehicle alone. Potassium antimonyl tartrate trihydrate added at the corresponding EC_50_ value (e.g. 0.02-0.03 mg/mL) was used as control drug.

Mouse macrophages (cell line J774) were cultivated in DMEM medium supplemented with 10% (v/v) FBS, 10 U/mL penicillin and 10 µg/mL streptomycin under a humidified 5% CO_2_/95 % air atmosphere at 37 ºC. Stock solutions of the test compounds were prepared as described for the anti-*T. b. brucei* assays and diluted in culture medium to obtain experimental concentrations from 100 to 0.01 µM. Each condition was tested in triplicate. Cell viability was evaluated using the WST-1 reagent (Roche) and EC_50_ values were obtained as essentially described in [4].

**References**

[1] Studier FW. (2005) Protein production by auto-induction in high-density shaking cultures. Protein Expr Purif. (2005); 41: 207-234.

[2] Haldane JBS . Enzymes. New York: Longmans, Green; 1930.

[3] Hirumi H., Hirumi K. Continuous cultivation of *Trypanosoma brucei* blood stream forms in a medium containing a low concentration of serum protein without feeder cell layers. J Parasitol. 1989; 75: 985-989.

[4] Demoro B, Sarniguet C, Sánchez-Delgado R, Rossi M, Liebowitz D, Caruso F, et al New organoruthenium complexes with bioactive thiosemicarbazones as co-ligands: potential antitrypanosomal agents. Dalton Trans. 2012; 41: 1534-1543.

[5] Maiwald F, Benítez D, Charquero D, Abad Dar M, Erdmann H, [Preu L](http://www.ncbi.nlm.nih.gov/pubmed/?term=Preu%20L%5BAuthor%5D&cauthor=true&cauthor_uid=24973661), et al. 9- and 11-substituted 4-azapaullones are potent and selective inhibitors of African trypanosome. Eur J Med Chem. 2014; 18: 274-283.
